# Impact of nitrogen fertilizer sustainability on corn crop yield: the role of beneficial microbial inoculation interactions

**DOI:** 10.1186/s12870-024-04971-3

**Published:** 2024-04-11

**Authors:** Fernando Shintate Galindo, Paulo Humberto Pagliari, Edson Cabral da Silva, Bruno Horschut de Lima, Guilherme Carlos Fernandes, Cassio Carlette Thiengo, João Victor Silva Bernardes, Arshad Jalal, Carlos Eduardo Silva Oliveira, Lucila de Sousa Vilela, Enes Furlani Junior, Thiago Assis Rodrigues Nogueira, Vagner do Nascimento, Marcelo Carvalho Minhoto Teixeira Filho, José Lavres

**Affiliations:** 1 College of Agricultural and Technological Sciences, Department of Crop Production, São Paulo State University, Dracena, 17900-000 Brazil; 2 Southwest Research and Outreach Center, Department of Soil, Water, and Climate, University of Minnesota, Lamberton, MN 56152 USA; 3 Department of Plant Health, Rural Engineering, and Soils, São Paulo State University, Ilha Solteira, 15345-000 Brazil; 4 Center for Nuclear Energy in Agriculture, University of São Paulo, Piracicaba, 13416-000 Brazil; 5 King Abdullah University of Science and Technology, Thuwal, 23955-6900 Kingdom of Saudi Arabia; 6 Department of Agricultural Sciences, São Paulo State University, Jaboticabal, 14884-900 Brazil

**Keywords:** *Azospirillum brasilense*, *Bacillus subtilis*, N synergists, N use efficiency and recovery, Biotic stress

## Abstract

**Background:**

Considering the challenges posed by nitrogen (N) pollution and its impact on food security and sustainability, it is crucial to develop management techniques that optimize N fertilization in croplands. Our research intended to explore the potential benefits of co-inoculation with *Azospirillum brasilense* and *Bacillus subtilis* combined with N application rates on corn plants. The study focused on evaluating corn photosynthesis-related parameters, oxidative stress assay, and physiological nutrient use parameters. Focus was placed on the eventual improved capacity of plants to recover N from applied fertilizers (AFR) and enhance N use efficiency (NUE) during photosynthesis. The two-year field trial involved four seed inoculation treatments (control, *A. brasilense*, *B. subtilis*, and *A. brasilense* + *B. subtilis*) and five N application rates (0 to 240 kg N ha^−1,^ applied as side-dress).

**Results:**

Our results suggested that the combined effects of microbial consortia and adequate N-application rates played a crucial role in N-recovery; enhanced NUE; increased N accumulation, leaf chlorophyll index (LCI), and shoot and root growth; consequently improving corn grain yield. The integration of inoculation and adequate N rates upregulated CO_2_ uptake and assimilation, transpiration, and water use efficiency, while downregulated oxidative stress.

**Conclusions:**

The results indicated that the optimum N application rate could be reduced from 240 to 175 kg N ha^−1^ while increasing corn yield by 5.2%. Furthermore, our findings suggest that replacing 240 by 175 kg N ha^−1^ of N fertilizer (-65 kg N ha^−1^) with microbial consortia would reduce CO_2_ emission by 682.5 kg CO_2_ ^−e^ ha^−1^. Excessive N application, mainly with the presence of beneficial bacteria, can disrupt N-balance in the plant, alter soil and bacteria levels, and ultimately affect plant growth and yield. Hence, highlighting the importance of adequate N management to maximize the benefits of inoculation in agriculture and to counteract N loss from agricultural systems intensification.

**Supplementary Information:**

The online version contains supplementary material available at 10.1186/s12870-024-04971-3.

## Introduction

Corn (*Zea mays* L.) is a globally consumed cereal, with a yearly production surpassing 1 billion tons [1]. Corn, along with wheat (*Triticum aestivum* L.), accounts for roughly 30% of all synthetic nitrogen (N) fertilizers consumed globally in crop production [2]. Inadequate application rates, broadcasting without incorporation, or excessively high rates on poorly drained soils are common examples of poor fertilizer management practices that frequently lead to low nitrogen use efficiency (NUE) and apparent fertilizer recovery (AFR) [3, 4]. The excessive use of fertilizers and other organic and inorganic amendments has been reported to present detrimental effects on water and soil quality on a global scale [5]. In this sense, the overapplication of N-based fertilizers has resulted in significant environmental issues, including water eutrophication, soil acidification, and biodiversity loss due to increased N losses [6, 7]. Several strategies have been developed to counteract the issues and diminish dependence on intensive chemical N fertilization in corn cultivation [8, 9].

In tropical regions, NUE and AFR become crucial, mainly due to the high N demand for corn plants [10, 11]. To enhance NUE and AFR under such conditions, it is fundamental to promote integrated N-fertilizer management strategies that prioritize plant growth and development. By adopting holistic approaches that optimize N utilization, including enhancing crop physiology (e.g. increasing photosynthetic parameters, C fixation, protein concentration, and plant biomass production) N absorption and corn crop productivity may be improved while minimizing the negative environmental consequences associated with excessive N application. Striking a balance between maximizing corn growth and development in contrast minimizing N-related environmental impacts is a key challenge that requires concerted efforts from researchers, farmers, and policymakers [12].

Crop production must be raised by 70% to meet the projected food demands in 2050 [12, 13]. This pressing challenge calls for integrated and coordinated efforts to preserve natural resources while intensifying agricultural practices [13]. The goal is to achieve higher crop yields without causing harmful impacts on the environment [14]. To address this critical issue, strategic and sustainable resource management is mandatory. Innovative agricultural practices that optimize the use of land, water, and nutrients to boost grain yield efficiently need to be employed [12]. By enhancing plant physiological processes and productivity through smart resource allocation, it is feasible to meet the expanding global food demand and avert the potential food security in the future decades [15].

Plant growth-promoting bacteria (PGPB) with multiple beneficial traits have shown great potential in improving various aspects of crop production under tropical conditions. Several studies have demonstrated that PGPB, such as *Azospirillum brasilense* and *Bacillus subtilis* can positively impact C fixation, NUE, AFR, and overall plant growth which could ultimately increase the yield of cereal crops [12, 16–19]. Research has also shown that *A. brasilense* enhances plant growth by boosting the production of several phytohormones (e.g. IAA, cytokinins, gibberellins) [20]. Furthermore, it enhances the proliferation of roots and thereby facilitates the acquisition of nutrients and water by the plants [21]. *Azospirillum brasilense* improves NUE from N-derived fertilizers [12, 18], the activity of nitrate reductase [22, 23], and plays a role in phosphate solubilization [24], among other beneficial effects. *Bacillus subtilis* is a well-known PGPB with both biofertilizer and biocontrol functions, widely employed in agricultural production [25]. *Bacillus subtilis* can promote plant growth through mechanisms such as regulation of plant hormones, stimulation of plant-induced systemic resistance (ISR), pathogen antagonism, and plant microbiome shaping [26]. Studies have demonstrated the potential of *B. subtilis* inoculation in different crop species, preventing plant diseases, reducing N losses by ammonia (N-NH_3_^+^) volatilization, runoff, and leaching, and increasing NUE [27–29]. To date, the additive hypothesis provides the most comprehensive explanation for the operating principles of *A. brasilense* and *B. subtilis* in promoting plant growth. According to this hypothesis, the multiple mechanisms of plant growth promotion mediated by these bacteria work in convergence or sequence, complementing each other to enhance the overall growth and performance of plants [17, 30, 31]. Nonetheless, most of the studies with PGPB are focused on the single effects of some bacteria instead of the benefits of microbial consortia (in our study jointing *A. brasilense* and *B. subtilis* in a co-inoculation). Also, studies evaluating the mechanistic effects of PGPBs on physiological and biochemical parameters associated with nutritional and biometric analyses aiming to explain changes in NUE and AFR are still scarce.

To our knowledge, this is one of the first studies that evaluated the microbial consortia with *A. brasilense* and *B. subtilis* coupled with N application rates focused on investigating the effects of these treatments on various aspects of corn plant physiology, biochemistry, and nutritional content, in a multi-approach study. Our primary objective was to determine if the inoculation and N application rates could enhance the corn plant’s ability to recover and utilize N from fertilizers more efficiently, ultimately leading to improved NUE and AFR. The goal was to identify strategies that could enhance NUE in corn crops, thereby reducing N losses to the environment and promoting more resource-efficient and environment-friendly farming practices. To achieve these goals, we conducted a comprehensive assessment of various physiological parameters, such as gas exchange parameters, biomass production, and leaf chlorophyll index. Additionally, we analyzed selected biochemical parameters such as hydrogen peroxide production (H_2_O_2_), lipid peroxidation (malondialdehyde - MDA), and soluble protein concentration, both related to oxidative stress in plants that impact C fixation.

## Materials and methods

### The experimental site, design, and treatments

A two-year field experiment was carried out at the São Paulo State University Experimental Station in the city of Selvíria, state of Mato Grosso do Sul, Brazil. The study site is coordinated at 20°22′ S and 51°22′ W, with an elevation of 335 m above sea level, had been used for cereal and legume crop cultivation for exceeding three decades. Over the past five years, the area has been under the management of a no-tillage agricultural system. Before corn cultivation, the crop sequence included corn and sorghum in succession. The study site climate was a dry winter tropical savannah characterized as Aw according to the Köppen-Geiger classification. Throughout the corn growth seasons of 2020/21 and 2021/2022 (November to March), daily measurements of rainfall and temperature were recorded from a weather station situated at the experimental site (Supplementary File 1). The soil in the experimental area is classified as clayey and specifically categorized as Rhodic Haplustox [32]. To assess the chemical attributes of the soil at a depth of 0–0.20 m, analysis was conducted following classical methods [33] (Supplementary File 2). The semi-micro Kjeldahl method was employed to ascertain the total N content in the soil [34] (Supplementary File 2). The concentrations of nitrate and ammonium (N-NO_3_^−^ and N-NH_4_^+^) in the soil were measured [35] (Supplementary File 2). Soil granulometry was determined using the pipette method outlined by Teixeira et al. [36].

The experiment consisted of a factorial design with four seed inoculation treatments and five N application rates. The four seed inoculation treatments included: control (without inoculation), single inoculation with *A. brasilense*, single inoculation with *B. subtilis*, and a co-inoculation with *A. brasilense* + *B. subtilis* (the microbial consortia applied in this study). The five N application rates ranged from 0 to 240 kg N ha^−1^ (0, 60, 120, 180 and 240 kg N ha^−1^ as urea source). The experiment was arranged in a randomized complete block design with four replications, resulting in a 4 × 5 scheme. Each experimental plot consisted of eight rows, each 0.50 m wide and 7 m in length. The operational area comprised the central six rows, excluding 1.0 m at the end of each cornrow, totaling 5 m within the working plots. The N application rates were derived from preceding studies concerning N management in corn cultivation in comparable tropical conditions [12, 18]. Urea (45% of N) was used as the N source and was applied at the V5/V6 phenological stage of the corn (five to six leaves completely unfolded). The seeds were inoculated with *A. brasilense* strains Ab-V5 and Ab-V6 (at a concentration of 2 × 10^8^ colony-forming units per milliliter (CFU mL^−1^), using the commercial liquid inoculant Azotrop®, Total Biotecnologia, Curitiba, PR, Brazil). Additionally, *B. subtilis* strains CCTB04 (at a concentration of 1 × 10^8^ CFU mL^−1^, using the commercial liquid inoculant Vult®, Total Biotecnologia, Curitiba, PR, Brazil) were employed. Commercial inoculants were chosen to represent real production conditions, allowing the use of inoculants that are readily available for purchase and use by farmers. These commercial inoculants have registration and quality control approval from the Brazilian Ministry of Agriculture and Livestock (MAPA). The aim of using commercial inoculants was to simulate practical production conditions, facilitating potential adoption by farmers for practical applications. The colony count of the liquid inoculant was conducted following the methodologies outlined by for *Bacillus* sp. [37]. and for *Azospirillum* sp. [38]. Inoculation was operated by mixing and coating the inoculants with corn seeds inside plastic kit bag, using a rate of 200 mL of liquid inoculant per hectare, immediately before sowing.

### Corn crop management

During the cultivation seasons of 2020/21 and 2021/22, a simple corn hybrid (Forseed® FS 587 PWU - POWERCORE™ ULTRA, LongPing High-Tech, Cravinhos, SP, Brazil) was seeded at a density of 7.0 viable seeds m^2^ using drill in a no-till system. A granular basal fertilization with an N-P_2_O_5_-K_2_O composition of 08-28-16 was applied to all treatments at corn sowing (at the rate of 350 kg ha^−1^). This basal fertilization was applied according to soil analysis and the specific requirements of the corn crop [39] and resulted in an application of 28 kg N ha^−1^ across the entire experimental area. Consequently, the total nitrogen applied in each treatment included the sum of the nitrogen applied at side-dress (ranging from 0 to 240 kg N ha^−1^) and the N applied during basal fertilization. Hence, the fertilizer application strategy involved two main steps: basal fertilization and side-dressing N fertilization. This approach is commonly practiced by cereal farmers in Brazil [12]. As we mentioned before, seed inoculation with *A. brasilense* and/or *B. subtilis* was performed at the time of sowing. The N-sidedress application, on the other hand, was done manually between the V4 and V6 phenological stages of corn. During this N application, the fertilizer was evenly distributed on the soil surface without incorporation. The corn crops were cultivated from November 12, 2020, to March 10, 2021, and from November 8, 2021, to March 7, 2022, for the 2020/21 and 2021/22 cropping seasons, respectively. The harvest was carried out 115 days after emergence for the first crop year and 116 days after emergence for the second crop year. To manage weeds, pre-and postemergence herbicides were used, while insect control followed the best practices for corn cultivation. The corn crops were grown under a rainfed system without any supplemental irrigation.

### Plant sampling and analysis

#### Samplings at the full flowering

During the flowering stage, seven corn plants (equivalent to 1m^2^) were harvested by cutting them at ground level to collect the shoots. In addition to the above-ground biomass samplings, the study also focused on the root system. To collect the roots, a lateral trench approximately 0.60 m deep, 1 m length and 0,50 m width was excavated within each plot. Particular attention was paid to positioning the trench in the center of the wide plant rows to enable the retrieval of a representative sample of the root system. Roots were sampled from this central position of the broad plant rows to ensure the comprehensive sampling of the entire root system. Following the sampling, the roots underwent a washing process using a 0.55-mm sieve for the removal of soil particles. Both the shoots and roots were then dried in a forced-air oven at 65 °C for 78 h. After drying, the shoot and root dry mass were sampled and converted to kg ha^−1^. Nitrogen concentration in shoot and root tissues was determined using the methodology involving the semi-micro Kjeldahl method with sulfur digestion analysis [40].

The leaf chlorophyll index (LCI) was determined indirectly by taking leaf readings at the main stem-leaf insertion using a portable non-destructive chlorophyll Falker meter (ClorofiLOG® -model CFL − 1030 Falker, Porto Alegre, RS, Brazil). For leaf gas exchange evaluations, non-destructive analyses were conducted using an infrared gas analyzer (LI-6400; LI-COR Inc., Lincoln, NE, USA). The measurements were taken under a photosynthetic photon flux density of 1,800 µmol m^−2^ s^−1^ and an air CO_2_ concentration of 380 µmol mol^−1^. The leaf temperature during the measurements ranged from 21 to 25 °C, following the methods used in previous studies [41–43]. The gas exchange parameters measured included CO_2_ assimilation rate expressed per unit area (A - µmol CO_2_ m^−2^ s^−1^), transpiration (E - µmol H_2_O m^−2^ s^−1^), stomatal conductance (gs - mol H_2_O m^−2^ s^−1^), and internal CO_2_ concentration in the substomatal chamber (Ci - µmol CO_2_ mol air^−1^). Biochemical analyses were performed to assess oxidative stress and protein content in the leaves. The concentration of hydrogen peroxide (H_2_O_2_) was determined following the method described by Alexieva [44]. Lipid peroxidation, represented by the concentration of malondialdehyde (MDA), was measured using the methodology of Heath and Packer [45]. The concentration of total soluble proteins was determined according to the Bradford assay [46].

### Samplings at the harvest time

The shoot (straw) biomass was determined using the same procedures mentioned before (section *Samplings at the full flowering*). After the corn plants reached maturity, the entire aboveground biomass, including leaves, stems, and any other aerial parts, were harvested and collected from each plot. After the corn plants reached maturity (phenological stage of R6), the grain yield was determined by collecting the spikes from the useful lines of each corn plot. Once collected, the spikes were mechanically threshed and weighed to measure the total grain yield of each plot. The grain yield was expressed in kg ha^−1^ and adjusted to a moisture content of 13% on a wet basis.

Nitrogen use efficiency (NUE - kg grain kg N applied^−1^) was calculated following the methodology presented in Eq. 1 [47].

1 $$NUE=\left(grain\,yield\,at\,Ny-grain\,yield\,at\,N0\right)\div N\,level\,applied$$

Apparent N-fertilizer recovery (AFR - %) was estimated based on methodology and presented in Eq. 2 [3]:

2 $$AFR=\left[\left(grain\,N\,acc.\,at\,Ny-grain\,N\,acc.\,at\,N0\right)\div N\,level\,applied\right]\times\,100$$

Physiological efficiency (PE - kg grain kg N accumulated^−1^) was presented in Eq. 3 [48]:

3 $$PE=\left(GY\,at\,Ny-GY\,at\,N0\right)\div(Plant\,N\,acc.\,at\,Ny-Plant\,N\,acc.\,at\,N0)$$

For all equations, Ny refers to the N level applied (N application rate) and N0 represents the absence of N application in side-dressing.

### Inorganic N concentration in shoot (straw) and soil after corn cultivation

The concentration of inorganic nitrogen (particularly nitrate - N-NO_3_^−^ and ammonium - N-NH_4_^+^) in the soil and shoot (straw) tissues was assessed using the procedure outlined Tedesco et al. [49]. For this analysis, 1 g of plant tissue (shoot) and 10 g of soil were extracted with 1 mol KCl L^−1^. The extracted samples were then distilled with MgO (for N-NH_4_^+^) and Devarda’s alloy and titrated with H_2_SO_4_ L^−1^ (for N-NO_3_^−^). Regarding the soil samples, they were collected and stored in a cold chamber at 4 °C until the inorganic N analysis was conducted. Total N was determined following the semi-micro Kjeldahl method [34]. The inorganic and total N analysis were performed on the treatments that contained the four inoculations at the N rate of 120 kg ha^−1^, which is the average N rate recommended for corn production in tropical regions.

### Statistical procedures and analysis

Initially, all data were assessed for homoscedasticity using Levene’s test (*p* ≤ 0.05). Following this, the data were examined for normality using the Shapiro-Wilk test, which demonstrated a normal distribution of the data (W ≥ 0.90). Subsequently, the data were subjected to an analysis of variance (F test) with repeated measures, considering cultivation years as repeated variables. For handling the repeated measurements within subjects, a compound symmetry model was utilized for the covariance parameters. Whenever a significant main effect or interaction was detected by the F test (*p* ≤ 0.05), additional comparisons were carried out using the Tukey test (*p* ≤ 0.05). Additionally, regression analysis was employed to determine whether a linear or nonlinear response to the N application rates occurred. The entire analytical process was performed using the ExpDes package in R software [50].

To identify dependent variables directly related to inoculation performance (contrasts between control – without inoculation and microbial consortia – *A. brasilense* + *B. subtilis*, both in response to 60–240 kg N ha^−1^), a Pearson correlation analysis (*p* ≤ 0.05) was performed and presented as a colored heatmap using the corrplot package using the functions corr and cor. mtest [50]. Principal component analysis (PCA) (also comparing the contrasts between control and microbial consortia) was performed using the FactoExtra and FactoMineR packages in R software following [12] procedures.

## Results

### Summary of analysis of variance

Supplementary file 3 contains the results from the analysis of variance highlighting the interactions between inoculations and N application rates that were found to be significant at the *p*-level ≤ 0.05. The remaining significant main effects and interactions are presented in Supplementary files 4–26. In the subsequent sections, are reported and discussed the significant interactions between inoculations and N application rates, exploring their implications for various physiological, biochemical, and agronomic parameters in corn.

### Physiological efficiency (PE), nitrogen use efficiency (NUE), and apparent N-fertilizer recovery (AFR)

**Fig. 1:**
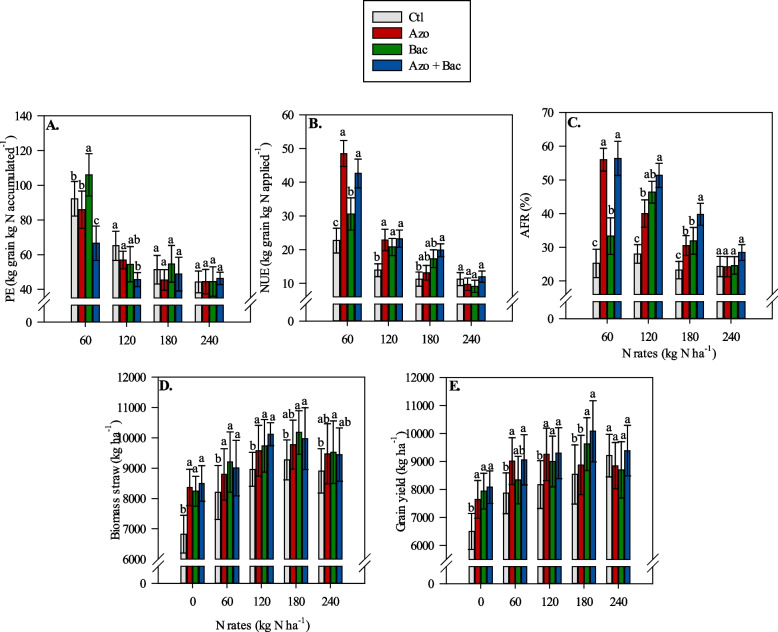
Interaction between inoculations and N rates on corn physiological efficiency (PE) (**A**), N use efficiency (NUE) (**B**), apparent fertilizer recovery (AFR) (**C**), biomass straw (**D**) and grain yield (**E**). Lowercase letters represent the difference between inoculations at each N rate; Ctl = control, Azo = single inoculation with *A. brasilense*, Bac = single inoculation with *B. subtilis* and Azo + Bac = co-inoculation with *A. brasilense* and *B. subtilis*; ± refers to standard deviation of the mean

Under low N application (60 kg N ha^−1^), PE was greater in treatments with single inoculation of *B. subtilis*. However, with 120 kg N ha^−1^ applied, the non-inoculated treatment showed greater PE than the co-inoculation treatment (Fig. 1A). Nitrogen use efficiency tended to be greater with both single and co-inoculation with *A. brasilense* and *B. subtilis* (Fig. 1B). Under low N application, single inoculation with *A. brasilense* and co-inoculation provided greater NUE compared to single inoculation with *B. subtilis* and the non-inoculated treatments. With 120 kg N ha^−1^, NUE was greater in single and co-inoculated treatments relative to the non-inoculated treatment (Fig. 1B). When 180 kg N ha^−1^ was applied, co-inoculation provided greater plant NUE compared to single inoculation and non-inoculated treatments (Fig. 1B). Similarly, AFR tended to increase with both single and co-inoculation with *A. brasilense* and *B. subtilis* (Fig. 1C). Under low N application, co-inoculation and single inoculation with *A. brasilense* provided greater AFR compared to single inoculation with *B. subtilis* and non-inoculated treatments. With 120 kg N ha^−1^, AFR was greater in co-inoculated treatments relative to single inoculation with *A. brasilense* and non-inoculated treatments. When 180 kg N ha^−1^ was applied, the microbial consortia provided greater AFR compared to single inoculations and non-inoculated treatments (Fig. 1C).

Physiological efficiency followed a non-linear response to N application rates, without reaching a point of maximum (plateau) within the N rates studied (Table 1). Meanwhile, NUE and AFR were found to decrease linearly with increasing N rates, except for AFR in the non-inoculated treatment, which was not statistically significant. Additionally, single inoculation with *B. subtilis* showed a response in AFR up to 178 kg N ha^−1^ (Table 1).

**Table 1:** Regression equations for physiological efficiency (PE), N use efficiency (NUE), apparent fertilizer recovery (AFR), biomass straw production, grain yield, shoot and root biomass, N shoot accumulation and leaf chlorophyll index (LCI) at flowering, as a function of N rates at each inoculation treatment

Treatment	PE (kg grain kg N accumulated^−1^)	NUE (kg grain kg N applied^−1^)
Control	Ŷ = 127.788–0.681x + 0.001 × ^2^ (R^2^ = 0.99^**^)	Ŷ = 24.027–0.061x (R^2^ = 0.78^**^)
Azo	Ŷ = 127.206–0.811x + 0.001 × ^2^ (R^2^ = 0.99^**^)	Ŷ = 55.073–0.209x (R^2^ = 0.86^**^)
Bac	Ŷ = 162.735–1.169x + 0.002 × ^2^ (R^2^ = 0.92^**^)	Ŷ = 36.429–0.113x (R^2^ = 0.97^**^)
Azo + Bac	Ŷ = 89.366–0.482x + 0.001 × ^2^ (R^2^ = 0.85^**^)	Ŷ = 48.238–0.158x (R^2^ = 0.90^**^)
**Treatment**	**AFR (%)**	**Biomass straw (kg ha**^**−1**^**)**
Control	ns	Ŷ = 6820.383 + 27.498x − 0.078 × ^2^ (R^2^ = 0.99^**^, P.M. = 176 kg N ha^−1^)
Azo	Ŷ = 63.887–0.174x (R^2^ = 0.96^**^)	Ŷ = 8260.360 + 15.089x − 0.050 × ^2^ (R^2^ = 0.94^**^, P.M. = 151 kg N ha^−1^)
Bac	Ŷ = 18.786 + 0.355x − 0.001 × ^2^ (R^2^ = 0.76^**^, P.M. = 178 kg N ha^−1^)	Ŷ = 8189.974 + 21.751x − 0.065 × ^2^ (R^2^ = 0.97^**^, P.M. = 167 kg N ha^−1^)
Azo + Bac	Ŷ = 67.825–0.158x (R^2^ = 0.97^**^)	Ŷ = 8354.989 + 20.693x − 0.066 × ^2^ (R^2^ = 0.89^**^, P.M. = 157 kg N ha^−1^)
**Treatment**	**Grain yield (kg ha**^**−1**^**)**	**Shoot biomass (kg ha**^**−1**^**)**
Control	Ŷ = 6838.649 + 10.141x (R^2^ = 0.92^**^)	Ŷ = 8350.770 + 14.818x − 0.040 × ^2^ (R^2^ = 0.94^**^, P.M. = 185 kg N ha^−1^)
Azo	Ŷ = 7784.245 + 19.991x − 0.067 × ^2^ (R^2^ = 0.86^**^, P.M. = 148 kg N ha^−1^)	Ŷ = 9043.568 + 14.631x − 0.045 × ^2^ (R^2^ = 0.99^**^, P.M. = 161 kg N ha^−1^)
Bac	Ŷ = 7772.477 + 17.476x − 0.053 × ^2^ (R^2^ = 0.79^**^, P.M. = 164 kg N ha^−1^)	Ŷ = 8835.659 + 24.406x − 0.078 × ^2^ (R^2^ = 0.96^**^, P.M. = 156 kg N ha^−1^)
Azo + Bac	Ŷ = 8052.723 + 19.374x − 0.055 × ^2^ (R^2^ = 0.90^**^, P.M. = 175 kg N ha^−1^)	Ŷ = 8981.098 + 22.565x − 0.070 × ^2^ (R^2^ = 0.95^**^, P.M. = 161 kg N ha^−1^)
**Treatment**	**Root biomass (kg ha**^**−1**^**)**	**N shoot accumulation (kg ha**^**−1**^**)**
Control	Ŷ = 561.259 + 3.559x − 0.009 × ^2^ (R^2^ = 0.97^**^, P.M. = 197 kg N ha^−1^)	Ŷ = 97.039 + 0.492x − 0.001 × ^2^ (R^2^ = 0.94^**^, P.M. = 189 kg N ha^−1^)
Azo	Ŷ = 741.384 + 4.250x − 0.015 × ^2^ (R^2^ = 0.96^**^, P.M. = 140 kg N ha^−1^)	Ŷ = 106.263 + 0.683x − 0.002 × ^2^ (R^2^ = 0.99^**^, P.M. = 171 kg N ha^−1^)
Bac	Ŷ = 626.585 + 4.447x − 0.012 × ^2^ (R^2^ = 0.97^**^, P.M. = 181 kg N ha^−1^)	Ŷ = 106.694 + 0.760x − 0.002 × ^2^ (R^2^ = 0.96^**^, P.M. = 158 kg N ha^−1^)
Azo + Bac	Ŷ = 820.312 + 4.575x − 0.017 × ^2^ (R^2^ = 0.96^**^, P.M. = 133 kg N ha^−1^)	Ŷ = 112.809 + 0.791x − 0.002 × ^2^ (R^2^ = 0.93^**^, P.M. = 152 kg N ha^−1^)
**Treatment**	**LCI**	
Control	Ŷ = 63.697 + 0.026x (R^2^ = 0.87^**^)	
Azo	Ŷ = 68.291 + 0.057x − 0.0001 × ^2^ (R^2^ = 0.95^*^, P.M. = 143 kg N ha^−1^)	
Bac	Ŷ = 65.450 + 0.085x − 0.0002 × ^2^ (R^2^ = 0.91^**^, P.M. = 143 kg N ha^−1^)	
Azo + Bac	Ŷ = 68.251 + 0.080x − 0.0002 × ^2^ (R^2^ = 0.96^**^, P.M. = 135 kg N ha^−1^)	

*Ctl* Control, *Azo* Single inoculation with *A. brasilense*, *Bac *Single inoculation with *B. subtilis* and Azo + Bac = co-inoculation with *A. brasilense* and *B. subtilis*; *ns* Not significant, *P.M.* the calculated point of maximum in equation (plateau)

** and *: significant at *p* < 0.01 and *p* < 0.05, respectively

### Corn biomass (straw) and grain yield

Corn biomass and grain yield increased with single and co-inoculation with *A. brasilense* and *B. subtilis* as N application rates increased (Fig. 1D, E). Without N supply and under low (60 kg N ha^−1^) and average (120 and 180 kg N ha^−1^) N application rates, the non-inoculated treatments tended to have lower biomass and grain yield compared to the single and co-inoculated treatments (Fig. 1D, E). In most cases, co-inoculation with *A. brasilense* + *B. subtilis* did not differ significantly from single inoculations in terms of corn biomass and grain yield. Under high N application rates (240 kg N ha^−1^), there were no significant differences between inoculated and non-inoculated treatments in terms of grain yield (Fig. 1E). Corn grain yield was negatively affected by the application of 240 kg N ha^−1^ as corn yield was on a downward trend with this rate.

Corn biomass and grain yield response to increasing N application rates were found to behave similarly across inoculation treatments. The maximum biomass and grain yield were observed at N application rates ranging from 148 to 176 kg N ha^−1^, depending on the specific inoculation treatment (Table 1). However, it is worth noting that for the non-inoculated treatment, a different response pattern for grain yield was observed. In this case, the correlation between N application rate and grain yield was best described by a linear increase. This result clearly shows how the use of PGPB can minimize the N requirement of corn plants.

### Plant biomass, N accumulation, and leaf chlorophyll index

**Fig. 2:**
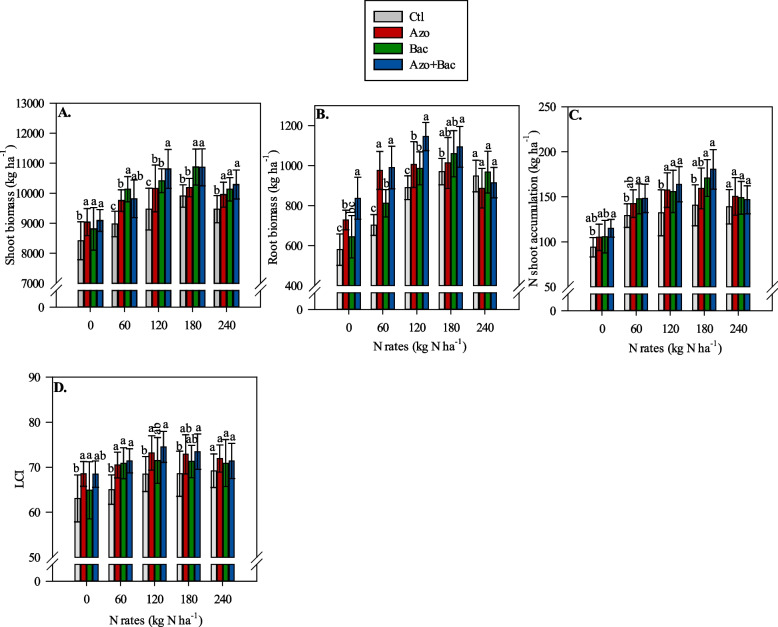
Interaction between inoculations and N rates on shoot (**A**) and root biomass (**B**), N shoot accumulation at flowering (**C**) and leaf chlorophyll index (LCI) (**D**). Lowercase letters represent the difference between inoculations at each N rate; Ctl = control, Azo = single inoculation with *A. brasilense*, Bac = single inoculation with *B. subtilis* and Azo + Bac = co-inoculation with *A. brasilense* and *B. subtilis*; ± refers to standard deviation of the mean

Figure 2A-D shows the general trends observed in shoot and root biomass, N shoot accumulation, and LCI under single and co-inoculation with *A. brasilense* and *B. subtilis*, along with N application rates. Shoot biomass and N shoot accumulation were higher in the inoculated plants (both single and co-inoculated) compared with non-inoculated plants (Fig. 2A, C). Similarly, LCI was greater with single inoculations of *A. brasilense* and *B. subtilis* than in the non-inoculated treatment (Fig. 2D). Root biomass increased with co-inoculation compared to single inoculation and non-inoculated treatment (Fig. 2B). Under low (60 kg N ha^−1^) and average (120 and 180 kg N ha^−1^) N application rates, the non-inoculated treatments tended to show decreased shoot and root biomass, N shoot accumulation, and LCI compared to the single and co-inoculated treatments (Fig. 2A-D). In most cases, co-inoculation with *A. brasilense* + *B. subtilis* did not differ significantly from single inoculations, except for shoot and root biomass when 120 kg N ha^−1^ was applied, where the microbial consortia provided the greatest values (Fig. 2A, B). Under high N application rates (240 kg N ha^−1^), no significant difference between inoculation treatments was observed for the abovementioned parameters.

The plant biomass, N accumulation, and LCI showed a positive response to increasing N rates, reaching their maximum values between 133 and 197 kg N ha^−1^, for the inoculated treatments. In contrast, a linear increase in LCI was observed as the N application rates increased for the non-inoculated treatment (Table 1).

The co-inoculated treatments exhibited significant improvements in root biomass and N shoot accumulation relative to the control treatment. Specifically, the co-inoculated treatments showed 44.3%, 40.9%, 28.8%, and 12.87% greater root biomass at N application rates of 0, 60, 120, and 180 kg ha^−1^, respectively, compared to the non-inoculated treatment. Similarly, the co-inoculated treatments showed 22.3%, 14.7%, 24.2%, and 28.6% greater N shoot accumulation at N application rates of 0, 60, 120, and 180 kg ha^−1^, respectively, relative to the non-inoculated treatment.

### Physiological analysis – gas exchange parameters

**Fig. 3:**
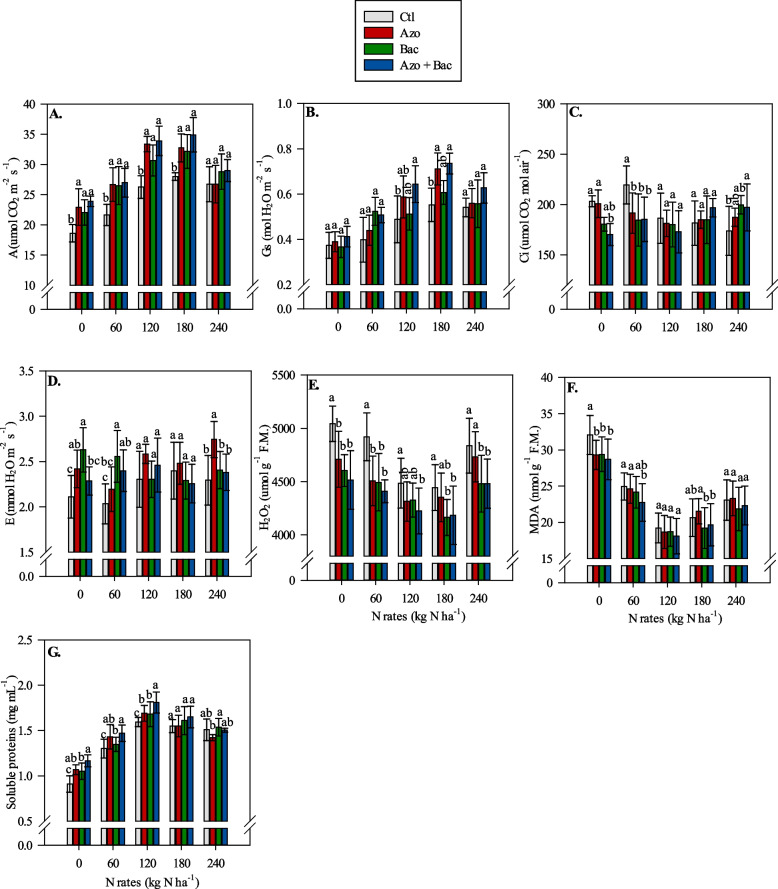
Interaction between inoculations and N rates on net photosynthetic rate (A) (**A**), stomatal conductance (Gs) (**B**), internal CO_2_ concentration in the substomatal chamber (Ci) (**C**), leaf transpiration (**D**), H_2_O_2_ (**E**), malondialdehyde (MDA) (**F**) and leaf soluble protein concentration (**G**). Lowercase letters represent the difference between inoculations at each N rate; Ctl = control, Azo = single inoculation with *A. brasilense*, Bac = single inoculation with *B. subtilis* and Azo + Bac = co-inoculation with *A. brasilense* and *B. subtilis*; ± refers to standard deviation of the mean

The CO_2_ assimilation rate (A) showed a trend of being higher in single and co-inoculated treatments relative to the non-inoculated treatment, particularly in the absence (0 kg N ha^−1^) and under low and average N application rates (60 to 180 kg N ha^−1^) (Fig. 3A). This indicates that the presence of *A. brasilense* and *B. subtilis*, either individually or together, upregulated the CO_2_ assimilation process, leading to improved photosynthetic activity in corn plants. Regarding Gs, its values were not significantly affected by inoculations, except when average N rates (120 and 180 kg N ha^−1^) were applied. In this case, the non-inoculated treatments showed lower Gs compared to the co-inoculation treatment with *A. brasilense* + *B. subtilis* (Fig. 3B). This suggests that the microbial consortia might have had a positive influence on stomatal regulation under conditions of moderate N application rates, potentially leading to more efficient water use and transpiration control. The values of Ci and E showed fluctuations across different inoculations. Without N application and at a low N rate (60 kg N ha^−1^) non-inoculated plants displayed higher Ci and lower E compared to the single and co-inoculated treatments (Fig. 3C, D). This may indicate that the microbial inoculation affected the gas exchange processes of the plants, potentially improving water-use efficiency and minimizing the loss of CO_2_.

### Biochemical analysis – oxidative stress parameters

The single and co-inoculation treatments with *A. brasilense* and *B. subtilis* typically resulted in decreased levels of reactive oxygen species (ROS) and lipid peroxidation, suggesting a reduction in oxidative stress and membrane damage in the corn plants. Specifically, H_2_O_2_ concentration tended to be lower in single and co-inoculated plants relative to non-inoculated plants (Fig. 3E). Similarly, MDA levels, a marker of lipid peroxidation and cellular damage, were lower in single and co-inoculated treatments compared to the non-inoculated treatment, particularly in the absence of N (Fig. 3F). Moreover, at the N rate of 60 kg ha^−1^, co-inoculated plants exhibited lower MDA levels relative to the non-inoculated treatment (Fig. 3F). Furthermore, at the N rate of 180 kg ha^−1^, co-inoculation with *A. brasilense* + *B. subtilis* and single inoculation with *B. subtilis* resulted in lower MDA levels compared to single inoculation with *A. brasilense* (Fig. 3F). This suggests that the microbial consortia of *A. brasilense* and *B. subtilis* were effective in reducing oxidative stress and lipid peroxidation even at average to high N application rates. The co-inoculated treatments showed a tendency to provide higher protein content compared to single inoculation and non-inoculated treatments at the N rate of 120 kg ha^−1^. The co-inoculated treatments also exhibited greater protein content than single inoculation with *B. subtilis* and non-inoculation at the N rate of 60 kg ha^−1^ (Fig. 3G). Soluble protein responded to the N application rate followed by a curvilinear response reaching their highest levels with N rates ranging from 160 to 175 kg ha^−1^, depending on the inoculation (Table 2). In contrast, H_2_O_2_ and MDA also showed a curvilinear response to the N application rate but never reached a plateau value (Table 2).

**Table 2:** Regression equations for corn net photosynthetic rate (A), stomatal conductance (Gs), internal CO_2_ concentration in the substomatal chamber (Ci), leaf transpiration (E), H_2_O_2_, malondialdehyde (MDA) and leaf soluble protein concentration as a function of N rates at each inoculation treatment

Treatment	A (µmol CO_2_ m^−2^ s^−1^)	Gs (mol H_2_O m^−2^ s^−1^)
Ctl	Ŷ = 17.931 + 0.099x − 0.0003 × ^2^ (R^2^ = 0.94^**^, P.M. = 165 kg N ha^−1^)	Ŷ = 0.373 + 0.0008x (R^2^ = 0.90^**^)
Azo	Ŷ = 21.937 + 0.150x − 0.0005 × ^2^ (R^2^ = 0.89^**^, P.M. = 150 kg N ha^−1^)	Ŷ = 0.354 + 0.003x − 0.000008 × ^2^ (R^2^ = 0.78^**^, P.M. = 188 kg N ha^−1^)
Bac	Ŷ = 22.454 + 0.063x − 0.0002 × ^2^ (R^2^ = 0.95^*^, P.M. = 158 kg N ha^−1^)	Ŷ = 0.377 + 0.002x − 0.000006 × ^2^ (R^2^ = 0.88^*^, P.M. = 167 kg N ha^−1^)
Azo + Bac	Ŷ = 22.715 + 0.143x − 0.0005 × ^2^ (R^2^ = 0.85^**^, P.M. = 143 kg N ha^−1^)	Ŷ = 0.389 + 0.003x − 0.000009 × ^2^ (R^2^ = 0.91^**^, P.M. = 167 kg N ha^−1^)
**Treatment**	**Ci (µmol CO**_**2**_**mol air**^**−1**^**)**	**E (mmol H**_**2**_**O m**^**−2**^**s**^**−1**^**)**
Ctl	Ŷ = 212.325–0.161x (R^2^ = 0.70^**^)	Ŷ = 2.082 + 0.001x (R^2^ = 0.59^**^)
Azo	ns	Ŷ = 2.297 + 0.001x (R^2^ = 0.53^**^)
Bac	ns	Ŷ = 2.668–0.004x + 0.00001 × ^2^ (R^2^ = 0.85^*^)
Azo + Bac	ns	ns
**Treatment**	**H**_**2**_**O**_**2**_**(µmol g**^**−1**^**F.M.)**	**MDA (nmol g**^**−1**^**F.M.)**
Ctl	Ŷ = 5126.517–8.250x + 0.028 × ^2^ (R^2^ = 0.78^**^)	Ŷ = 32.195–0.162x + 0.0005 × ^2^ (R^2^ = 0.98^**^)
Azo	Ŷ = 4743.616–6.8305x + 0.027 × ^2^ (R^2^ = 0.93^**^)	Ŷ = 29.616–0.128x + 0.0004 × ^2^ (R^2^ = 0.91^**^)
Bac	Ŷ = 4651.653–5.050x + 0.017 × ^2^ (R^2^ = 0.73^**^)	Ŷ = 29.775–0.1368x + 0.0004 × ^2^ (R^2^ = 0.97^**^)
Azo + Bac	Ŷ = 4557.840–5.025x + 0.018 × ^2^ (R^2^ = 0.81^**^)	Ŷ = 28.816–0.138x + 0.0004 × ^2^ (R^2^ = 0.98^**^)
**Treatment**	**Soluble proteins (mg mL**^**−1**^**)**	
Ctl	Ŷ = 0.911 + 0.008x − 0.000025 × ^2^ (R^2^ = 0.98^**^, P.M. = 160 kg N ha^−1^)	
Azo	Ŷ = 1.069 + 0.008x − 0.000025 × ^2^ (R^2^ = 0.96^**^, P.M. = 160 kg N ha^−1^)	
Bac	Ŷ = 1.034 + 0.007x − 0.00002 × ^2^ (R^2^ = 0.96^**^, P.M. = 175 kg N ha^−1^)	
Azo + Bac	Ŷ = 1.148 + 0.008x − 0.000025 × ^2^ (R^2^ = 0.93^**^, P.M. = 160 kg N ha^−1^)	

*Ctl* Control, *Azo* Single inoculation with *A. brasilense*, *Bac* Single inoculation with *B. subtilis* and Azo + Bac = co-inoculation with *A. brasilense* and *B. subtilis*; *ns* Not significant, *P.M.* the calculated point of maximum in equation (plateau)

** and *: significant at *p* < 0.01 and *p* < 0.05, respectively

The co-inoculated treatments exhibited higher soluble protein concentrations compared to the non-inoculated treatment at different N application rates. Specifically, co-inoculated treatments showed 28.6%, 13.1%, and 13.8% greater soluble protein concentrations at N rates of 60, 120, and 180 kg ha^−1^, respectively, relative to the non-inoculated treatment (Fig. 3G).

### Inorganic and total nitrogen content in straw and soil after corn cultivation

**Fig. 4:**
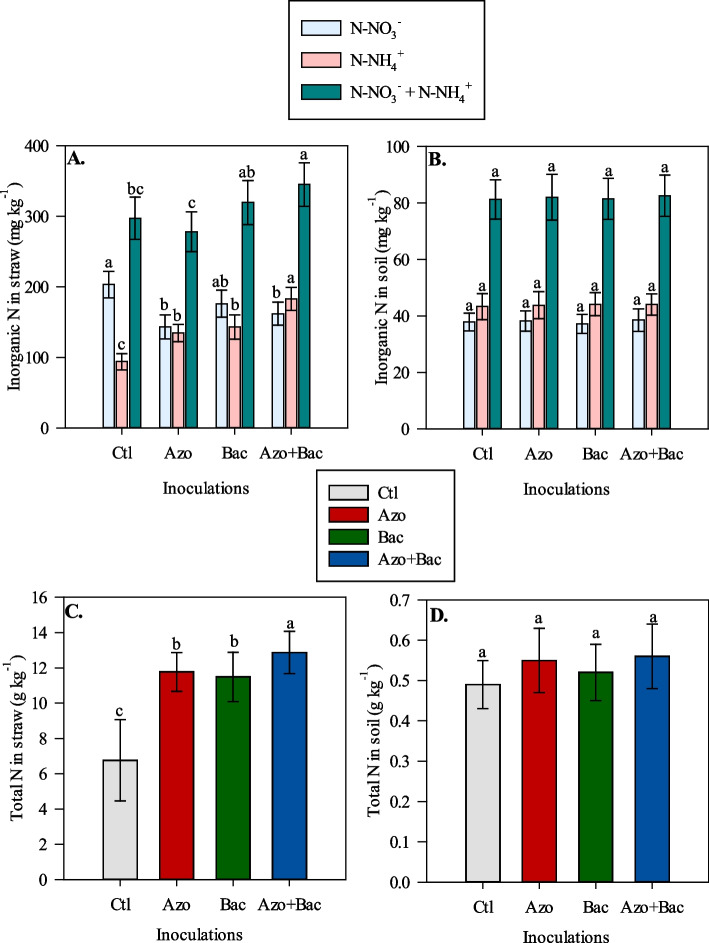
Nitrate (NO_3_^−^), ammonium (NH_4_^+^), inorganic (NO_3_^−^ + NH_4_^+^) in straw (**A**) and soil (**B**) and total N concentrations in straw (**C**) and soil (**D**) affected by inoculations. Ctl = control, Azo = single inoculation with *A. brasilense*, Bac = single inoculation with *B. subtilis* and Azo + Bac = co-inoculation with *A brasilense* and *B. subtili*s; ± refers to standard deviation of the mean

The analysis of straw N-NO_3_^−^ and N-NH_4_^+^ content showed distinct responses among the different inoculation treatments. Straw N-NO_3_^−^, was found to be greater in the non-inoculated treatment compared to single inoculation with *A. brasilense* and co-inoculation with A. *brasilense* + *B. subtilis* (Fig. 4A). In contrast, N-NH_4_^+^ content in the straw was greater in co-inoculated treatments compared to the single inoculations with *A. brasilense*, *B. subtilis*, and non-inoculated treatments (Fig. 4A). This result shows that the co-inoculation with *A. brasilense* + *B. subtilis* resulted in higher levels of N-NH_4_^+^ and led to a reduction in N-NO_3_^−^ content in corn shoots relative to other treatments. Additionally, the straw inorganic N content, which comprises both N-NO_3_^−^ and N-NH_4_^+^, followed a similar trend as observed for N-NH_4_^+^. Co-inoculated treatments showed higher levels of straw inorganic N compared to single inoculation with *A. brasilense* and the non-inoculated treatments (Fig. 4A). Similarly, co-inoculated treatments showed higher levels of total N in straw compared to single inoculations and non-inoculated treatments (Fig. 4C). Concerning soil N content, no significant differences were observed among the different inoculation treatments for inorganic and total N content (Fig. 4B and D).

### Pearson’s correlation (Heatmap) and principal component analysis (PCA) as a function of inoculations and N application rates

**Fig. 5:**
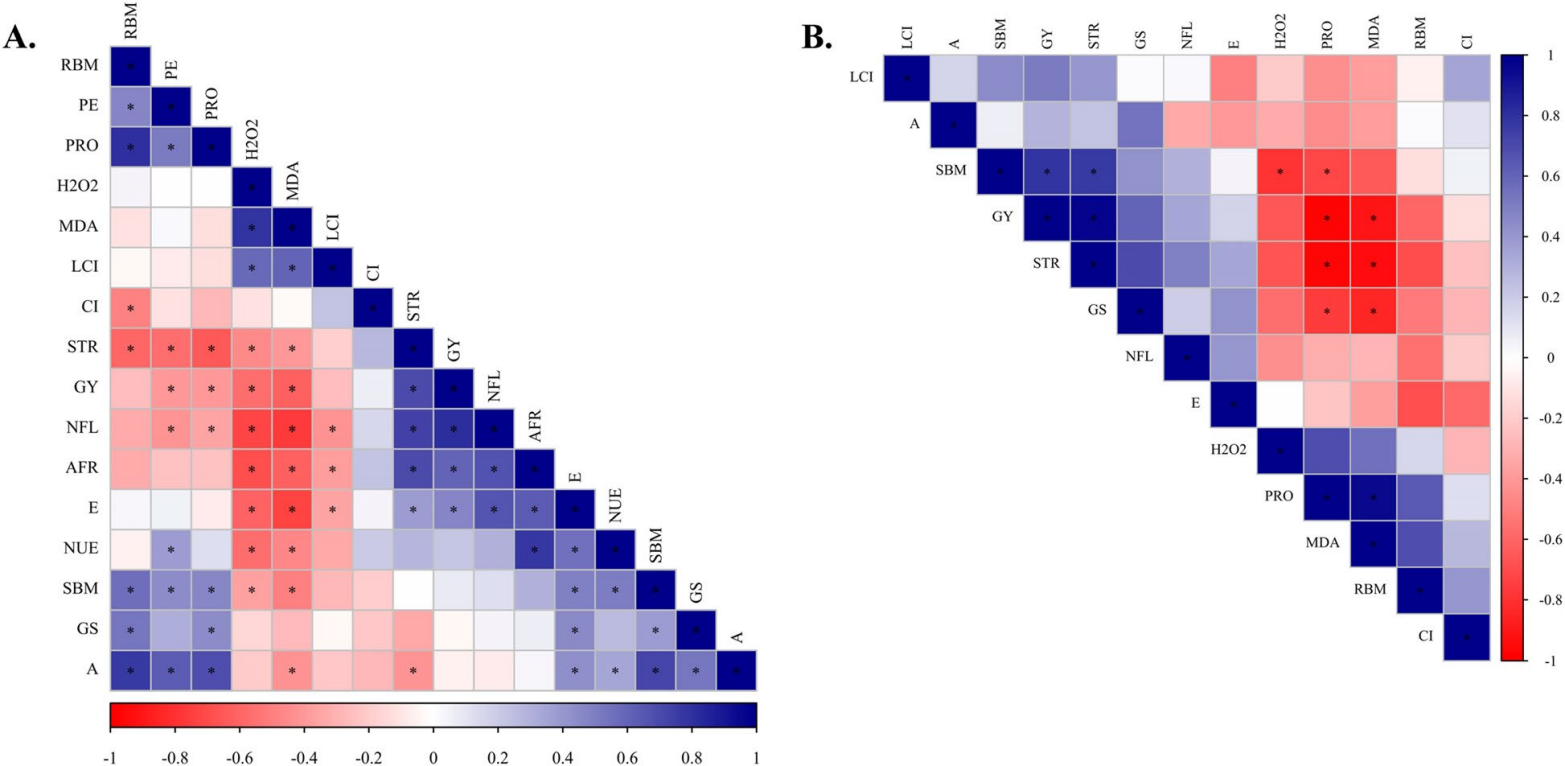
Heatmap of the Pearson correlation coefficients obtained from variables analyzed in corn without inoculation (control treatment) in response to N rates with (60 to 240 kg N ha^−1^) (**A**) and without N application (**B**). *Indicates significant correlation (*p* < 0.05); shoot biomass = SBM, root biomass = RBM, N shoot accumulation at flowering = NFL, leaf chlorophyll index = LCI, net photosynthetic rate = A, stomatal conductance = GS, internal CO_2_ concentration in the substomatal chamber = CI, transpiration = E, H2O2 = H_2_O_2_, malondialdehyde = MDA, leaf soluble proteins = PRO, straw production = STR, grain yield = GY, physiological efficiency = PE, N use efficiency = NUE, apparent fertilizer recovery = AFR

**Fig. 6:**
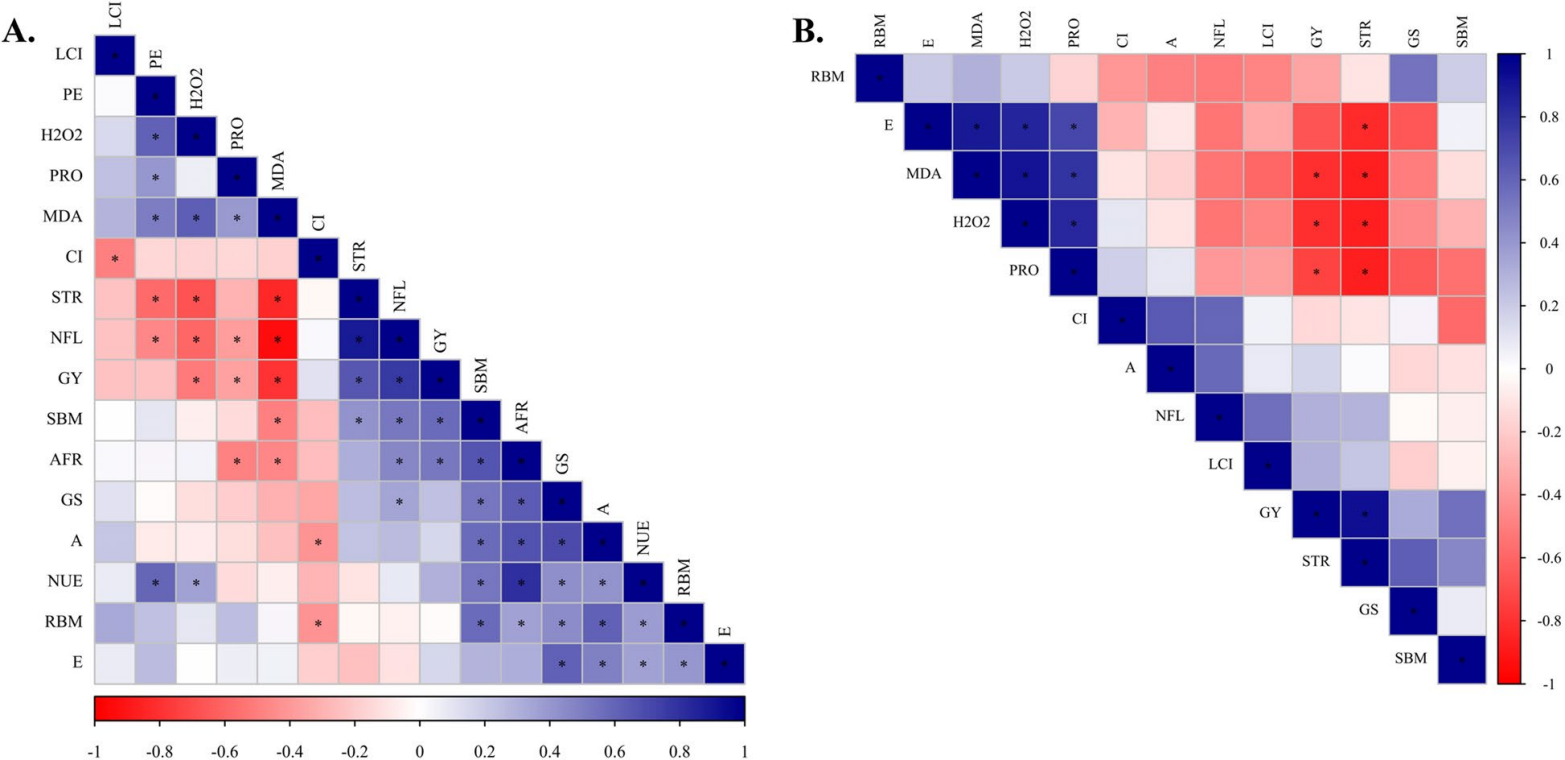
Heatmap of the Pearson correlation coefficients obtained from variables analyzed in corn with *A. brasilense* and *B. subtilis* inoculation in response to N rates with (60 to 240 kg N ha^−1^) (**A**) and without N application (**B**). *Indicates significant correlation (*p* < 0.05); shoot biomass = SBM, root biomass = RBM, N shoot accumulation at flowering = NFL, leaf chlorophyll index = LCI, net photosynthetic rate = A, stomatal conductance = GS, internal CO_2_ concentration in the substomatal chamber = CI, transpiration = E, H_2_O_2_ = H_2_O_2_, malondialdehyde = MDA, leaf soluble proteins = PRO, straw production = STR, grain yield = GY, physiological efficiency = PE, N use efficiency = NUE, apparent fertilizer recovery = AFR

Figures 5 and 6 present Pearson’s linear correlations in the form of a heatmap depicting the relationships among the evaluated parameters for non-inoculated (without inoculation) (Fig. 5) and co-inoculation with *A. brasilense* + *B. subtilis* treatments (Fig. 6). This visualization allows for a comprehensive understanding of the interplay between the contrasting inoculations and their impacts on the physiological, biochemical, nutritional, and productive parameters measured.

Analyzing the Pearson’s correlations in non-inoculated plots coupled with N application rates (60 to 240 kg N ha^−1^), it is observed that grain yield showed negative correlations with physiological efficiency, soluble proteins, H_2_O_2,_ and malondialdehyde concentrations (Fig. 5). In contrast, grain yield showed positive correlations with N shoot accumulation at flowering, apparent fertilizer recovery, and transpiration (Fig. 5). In the absence of N fertilization, grain yield showed negative correlations with soluble proteins and malondialdehyde concentration and positive correlations with shoot biomass at flowering and biomass yield (Fig. 5).

Differently, in microbial consortia plots coupled with N application rates, grain yield showed negative correlations with H_2_O_2_, soluble proteins, and malondialdehyde concentrations and positive correlation with shoot biomass at flowering, N shoot accumulation at flowering, biomass yield, and apparent fertilizer recovery (Fig. 6). In the absence of N fertilization, grain yield presented negative correlations with H_2_O_2_, soluble proteins, and malondialdehyde concentrations and a positive correlation with biomass yield (Fig. 6).

**Fig. 7:**
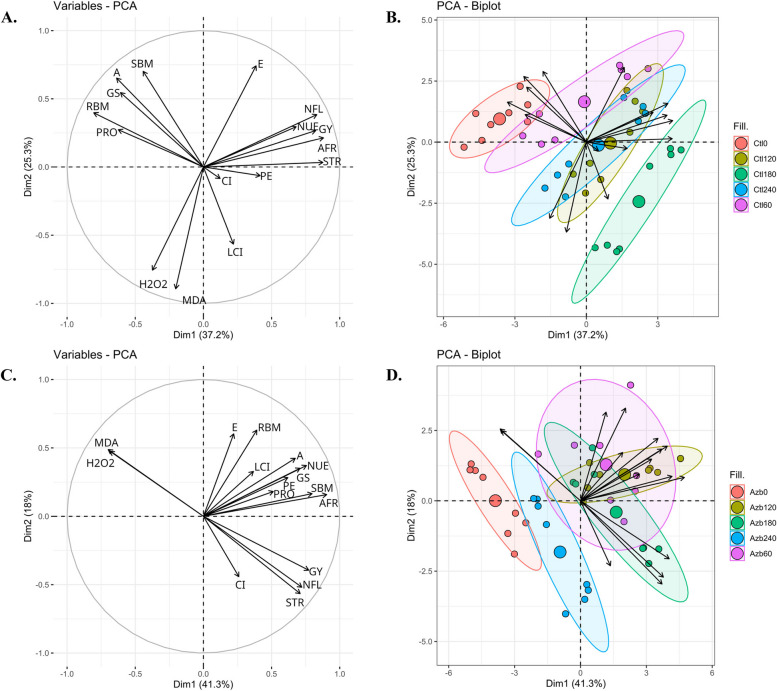
Loadings and biplot graphics of principal component analysis among the relationship between corn shoot (SBM) and root biomass (RBM), N shoot accumulation at flowering (NFL), leaf chlorophyll index (LCI), net photosynthetic rate (A), stomatal conductance (GS), internal CO_2_ concentration in the substomatal chamber (CI), transpiration (E), H_2_O_2_ (H2O2), malondialdehyde (MDA), leaf soluble proteins (PRO), straw production (STR), grain yield (GY), physiological efficiency (PE), N use efficiency (NUE), apparent fertilizer recovery (AFR) evaluated in the control (**A** and **B**) and *A. brasilense* + *B. subtilis* inoculated treatments (**C** and **D**). Ctl0, Ctl60, Ctl120, Ctl180 and Ctl240 refers to the absence of inoculation (control) associated with 0, 60, 120, 180 and 240 kg N ha^−1^ applied in side-dressing. Azb0, Azb60, Azb120, Azb180 and Azb240 refers to *A. brasilense* + *B. subtilis* inoculated treatments associated with 0, 60, 120, 180 and 240 kg N ha^−1^ applied in side-dressing

The eigenvalues of the four principal components extracted were greater than 1, indicating that these components can be grouped into a four-component model. This model accounts for 80.1% and 78.6% of the data variation in the non-inoculated and microbial consortia plots, respectively (Table 3; Fig. 7). In the non-inoculated plots, PC1 accounted for 37.2% of the variance, demonstrating a positive correlation between N shoot accumulation at flowering, biomass, grain yield, N use efficiency, and apparent fertilizer recovery (Table 3; Fig. 7). PC 2 exhibited a positive correlation among shoot biomass, net photosynthetic rate, and transpiration (Table 3; Fig. 7). Conversely, productions of H_2_O_2_ and malondialdehyde were negatively correlated with the aforementioned components of PC2 (Table 3; Fig. 7). PC1 and PC2 together accounted for 62.5% of the cumulative variance (Table 3; Fig. 7). The remaining two extracted factors can be considered negligible in terms of both explained variability and eigenvalues (Table 3; Fig. 7). In microbial consortia plots, PC1 represented 41.3% of the variance, indicating a positive correlation among shoot biomass, N shoot accumulation at flowering, net photosynthetic rate, stomatal conductance, biomass, grain yield, N use efficiency, and apparent fertilizer recovery (Table 3; Fig. 7). However, concentrations of H_2_O_2_ and malondialdehyde were negatively correlated with the aforementioned components of PC1 (Table 3; Fig. 7). PC2 exhibited a positive correlation between root biomass and transpiration (Table 3; Fig. 7). PC3 demonstrated a positive correlation among internal CO_2_ concentration in the substomatal chamber, soluble protein, and physiological efficiency (Table 3; Fig. 7). PC1, PC2, and PC3 collectively represented 71.7% of the cumulative variance (Table 3; Fig. 7). The remaining extracted factor (PC4) can be considered negligible in terms of both explained variability and eigenvalue (Table 3; Fig. 7).

**Table 3:** Factor loadings of a principal component analysis for corn crop in control and microbial consortia treatments; bold loadings > 0.3

	Without inoculation (control)			
Parameters	PC1	PC2	PC3	PC4
SBM	-0.18	**0.34**	-0.002	0.13
RBM	-0.29	0.19	0.19	-0.09
NFL	**0.34**	0.19	-0.10	-0.14
LCI	0.09	-0.28	0.16	0.23
A	-0.26	**0.32**	0.13	0.10
Gs	-0.24	0.27	-0.06	0.02
Ci	0.05	-0.04	**-0.33**	**0.84**
E	0.15	**0.36**	0.01	0.06
H2O2	-0.15	**-0.37**	0.14	-0.06
MDA	-0.08	**-0.44**	0.15	0.02
PRO	-0.25	0.13	**0.39**	0.07
STR	**0.35**	0.01	-0.04	-0.08
GY	**0.33**	0.13	-0.06	-0.22
PE	0.17	-0.03	**0.61**	0.09
NUE	**0.31**	0.14	**0.40**	0.27
AFR	**0.36**	0.10	0.20	0.07
Variance (%)	37.2	25.3	11.2	6.4
Cumulative variance (%)	37.2	62.5	73.7	80.1
Eingenvalues	5.95	4.05	1.79	1.03
	**With *A. brasilense +*** ***B. subtilis*** **inoculation**			
Parameters	PC1	PC2	PC3	PC4
SBM	**0.31**	0.09	-0.19	0.07
RBM	0.15	**0.37**	-0.11	-0.19
NFL	**0.31**	-0.29	-0.21	-0.01
LCI	0.14	0.19	0.22	**-0.62**
A	**0.32**	0.25	-0.23	-0.11
Gs	**0.30**	0.20	-0.23	0.05
Ci	0.10	-0.26	**0.38**	**0.41**
E	0.08	**0.35**	-0.19	**0.34**
H2O2	**-0.36**	0.28	-0.08	0.19
MDA	**-0.37**	0.28	0.02	0.14
PRO	0.19	0.10	**0.50**	-0.15
STR	**0.32**	-0.29	-0.15	-0.18
GY	**0.31**	-0.23	-0.11	0.20
PE	0.24	0.16	**0.47**	0.14
NUE	**0.32**	0.21	0.14	0.24
AFR	**0.35**	0.09	0.04	0.13
Variance (%)	41.3	18.0	12.4	6.9
Cumulative variance (%)	41.3	59.3	71.7	78.6
Eingenvalues	6.61	2.88	1.99	1.10

*SBM* Shoot biomass, *RBM* Root biomass, *NFL* N shoot accumulation at flowering, *LCI* Leaf chlorophyll index, *A* net photosynthetic rate, *GS* Stomatal conductance, *CI* Internal CO_2_ concentration in the substomatal chamber, *E* Transpiration, *H2O2* H_2_O_2_, *MDA* Malondialdehyde, *PRO* Leaf soluble proteins, *STR* Straw production, *GY* Grain yield, *PE* Physiological efficiency, *NUE* N use efficiency, *AFR* Apparent fertilizer recovery

## Discussion

### Effect of inoculations coupled with N management in corn growth and development

Our results suggest that the combined effects of microbial consortia and N application rates play a crucial role in enhancing N use efficiency (NUE) and apparent fertilizer recovery (AFR) leading to an increased plant N accumulation, leaf chlorophyll index (LCI), shoot and root biomass, and corn grain yield. Co-inoculation generally showed comparable results to single inoculations, demonstrating the potential benefits of using PGPBs in corn production systems to improve overall nitrogen use and management and crop performance. The positive Pearson’s correlation between GY × SBM, GY × NFL, and GY × AFR; SBM × AFR, SBM × NUE, and SBM × RBM in co-inoculated treatments coupled with N application rates (60 to 240 kg N ha^−1^) strengthen this hypothesis (Fig. 6A). Similar positive results related to single or co-inoculation with *A. brasilense* and *B. subtilis* have been reported in different crops [12, 18, 19, 28, 29, 51–53]. Although recent papers have been exploring the benefits of PGPBs in plant growth and nutrient use efficiency, to our knowledge, this is one of the first studies focused on understanding how the microbial consortia with *A. brasilense* and *B. subtilis* affect N use efficiency and recovery in field-grown corn plants. To achieve this, we used multitiered physiological, biochemical, and nutritional approaches at leaf, plant, and soil levels, therefore focusing on plant responses.

As we mentioned before, the results of this study demonstrate the positive impact of co-inoculation with *A. brasilense* and *B. subtilis* on NUE and AFR in corn cultivation. For instance, compared to the non-inoculated treatment, the co-inoculated plots showed substantial improvements in both NUE and AFR at different N application rates. Specifically, the co-inoculated plots exhibited 88%, 68%, and 76% greater NUE at N rates of 60, 120, and 180 kg N ha^−1^, respectively (Fig. 1). This indicates that the microbial consortia promoted better utilization of the applied N, resulting in higher grain yield per unit of N applied. Similarly, the co-inoculated plots showed significant improvements in AFR, with 123%, 83%, and 72% greater AFR at N application rates of 60, 120, and 180 kg N ha^−1^, respectively (Fig. 1). This indicates that a larger proportion of the applied N was effectively absorbed and used by the corn plants in the co-inoculated plots compared to non-inoculated treatments. Interestingly, the results of the physiological efficiency (PE) pointed out that the co-inoculated treatments provided a reduction in PE. Thus, while the co-inoculated plots showed improved N uptake and utilization, this did not translate into a proportional increase in grain yield. This phenomenon could be attributed to diverse factors affecting the overall efficiency of N conversion in corn hybrids. One possible explanation could be the limitation of other essential nutrients or environmental factors that may have hindered the corn plant’s ability to fully utilize the greater N uptake. Although one of the most important, N is just one of the many essential elements required for optimal plant growth and productivity. If other nutrients, such as P, K, or micronutrients were limiting, the plant’s physiological processes might not have been fully optimized, leading to a lower PE [54]. Additionally, environmental factors like water availability, temperature, and light intensity play a key role in nutrient uptake and utilization [55]. If any of these factors were suboptimal during the growing season, it could have affected the ability of the corn plants to convert the additional N into productive biomass efficiently. In addition, is also valid to mention that not all additional N provided to the plant will be specifically converted in grain production since there are several physiological processes involved in plant growth that not specifically result in grain production.

Overall, under high N application rates (240 kg N ha^−1^), there were no significant differences between inoculation treatments in terms of NUE, AFR, biomass production, and grain yield (Fig. 1). This suggests that at higher N application rates, the influence of inoculation with *A. brasilense* and *B. subtilis* on corn growth was not as pronounced, possibly because the high N levels provided sufficient N for the crop, minimizing the additional benefits from the microbial consortia. Our regression equations showed that the maximum response to N application rates in the most relevant parameter studied varied between 133 - 188 kg N ha^−1^ when inoculations were performed (Tables 1 and 2). These findings indicate that there is an optimal range of N application that maximizes plant growth and N accumulation and that the presence of PGPBs, either singly or in combination, can enhance these responses. Furthermore, when higher amounts of N than required for optimum plant growth are applied (e.g. 240 kg N ha^−1^) plants will be less effective at producing grains because they will spend more energy to produce biomass. The higher biomass being produced likely will waste resources and result in lower grain production. The results of this study clearly show that when single or co-inoculation is performed over the application of N will have a negative impact on corn crop performance.

The higher sensitivity of treatments containing *A. brasilense* (single inoculation or co-inoculation) to excessive N rates compared to *B. subtilis* suggests that *B. subtilis* may be more involved in promoting plant growth through mechanisms other than solely biological N fixation (BNF) or direct acquisition of N. As an example, it may enhance nutrient uptake efficiency, stimulate systemic resistance against pathogens, or influence plant hormone regulation, leading to improved growth and overall plant health conferring abiotic and biotic stress tolerance to plants by biofilm formation, induced systemic resistance and lipopeptide production [56]. Nonetheless, *B. subtilis* was previously reported to enhance BNF, mitigate N-NH_3_^+^ emissions to the atmosphere shifting soil N cycling microbiomes, slowing nitrification and enhancing denitrification in soil, thus reducing N losses [28, 29, 57]. In addition, *A. brasilense* is known for its ability to promote plant growth through various mechanisms, including the production of phytohormones like auxins, gibberellins, and cytokinins, which can stimulate root development and N uptake [20]. Inoculation with *A. brasilense* also can enhance BNF, nitrate reductase activity, and solubilize phosphates, further enhancing nutrient availability to the plant [17]. The recent meta-analysis conducted by Barbosa et al. [58] corroborates with the verified benefits of corn inoculation with *A. brasilense* strains Ab-V5 and Ab-V6. The meta-analysis included data from 60 studies, comprising 103 field trials conducted in 54 different locations across Brazil. The results indicated that inoculation with these *A. brasilense* strains resulted in an average increase of 5.4% in grain yield. One interesting finding from the meta-analysis is that the benefits of inoculation were more pronounced under lower N rates (≤ 50 kg ha^−1^), where the grain yield increase was 8%. In contrast, at higher N rates (> 200 kg ha^−1^), the grain yield increase from inoculation was 3.8%. This implies that the beneficial impacts of *A. brasilense* inoculation on corn growth and yield are particularly pronounced when N availability in soil is restricted. When excessive N is applied to the soil, it may lead to an imbalance in nutrient uptake and utilization, which can negatively impact plant growth. In this scenario, the additional N application may interfere with the plant’s hormonal balance and nutrient acquisition processes, resulting in the verified reduced PE and grain yield.

### Mechanisms underlying *A. brasilense*and *B. subtilis* inoculation in enhancing nitrogen use efficiency (NUE) and recovery key pathways

The mechanism of microbial consortia inoculation in improving N use efficiency and recovery was investigated at three levels. The first level was related to nutritional and biometric parameters. The observed increase in root biomass and N accumulation due to the use of microbial consortia appears to be one of the most important mechanisms driving the improvements in NUE and AFR, ultimately leading to enhanced corn growth and grain yield. This positive correlation among root biomass and some physiological parameters (RBM × NUE, RBM × AFR, RBM × A, and RBM × Gs) reinforces the hypothesis that the benefits of PGPBs on root development play a crucial role in promoting overall plant performance (Fig. 6A). Several studies have reported the positive effects of PGPBs on lateral root growth and root hairs in various crops [59–61]. These beneficial effects can alter plant physiology and root architecture, enabling plants to explore and penetrate the soil more effectively, leading to increased water and nutrient uptake [62, 63]. A robust root system not only facilitates nutrient uptake but also leads to increased deposition of organic C and N into the soil. This enhanced rhizo-deposition creates a more favorable environment for rhizosphere biodiversity by providing a nutrient-rich substrate for microbial activity [64]. Accordingly, this forwards the development of a diverse microbial community, which in turn contributes to nutrient cycling, disease suppression, and overall soil health. Moreover, the increased organic C and N inputs from the roots stimulate microbial activity and nutrient mineralization, further enhancing soil fertility and supporting overall plant functions such as growth, resilience to stress, and productivity. Also, the greater N accumulation observed in corn plants, notably as N-NH_4_^+^ form, alongside unchanged inorganic and total N dynamics in the soil following corn cultivation, can be attributed to several contributing factors. Firstly, the use of *Azospirillum* sp. in the microbial consortia may contribute to BNF, leading to the production of NH_4_^+^ from atmospheric N_2_ [17, 65]. Secondly, *Bacillus* sp. may play a role in delaying the soil nitrification process, temporarily increasing the availability of NH_4_^+^ in the soil and subsequently in corn plants [28, 29]. Additionally, the benefits on nitrate reductase activity related on literature, would potentially stimulate root development supported by co-inoculation, and could lead to enhanced conversion of N-NO_3_^−^ to N-NH_4_^+^ in plants [22].

The second level was physiological parameters. Overall, the results suggest that the presence of *A. brasilense* and *B. subtilis*, especially in combination, could positively influence physiological parameters related to photosynthesis and water use in corn plants, particularly under conditions of limited N availability. The co-inoculated treatments consistently exhibited improved A and Gs relative to the control treatment across N-application rates. Specifically, the co-inoculated treatments showed 28.5%, 25.0%, 28.9%, and 24.6% higher CO_2_ assimilation rates at N rates of 0, 60, 120, and 180 kg N ha^−1^, respectively, relative to the control (Fig. 3). This result indicates that the microbial consortia of *A. brasilense* and *B. subtilis* positively influenced the ability of corn plants to assimilate CO_2_ and perform photosynthesis, leading to increased C fixation in corn plants, and enhancing root and shoot growth. Additionally, the co-inoculated treatments exhibited 10.8%, 30.7%, 33.3%, and 34.5% higher Gs at N rates of 0, 60, 120, and 180 kg N ha^−1^, respectively, compared to the non-inoculated treatment (Fig. 3). Therefore, we can assume the presence of *A. brasilense* and *B. subtilis*, especially in combination, enhanced the stomatal conductance of corn plants. Stomatal conductance plays a crucial role in regulating water loss through transpiration and facilitating the exchange of CO_2_ during photosynthesis [66]. The higher Gs verified in co-inoculated treatments may indicate that the microbial inoculations positively influenced water use efficiency and the ability of corn plants to adapt to different N application rates.

The third evaluated level was biochemical analysis related to oxidative stress due to reactive oxygen species (ROS). Our study showed that microbial consortia generally led to lower levels of H_2_O_2_ and MDA coupled with increased soluble protein concentration (Fig. 3), indicating reduced oxidative stress and membrane damage in the corn plants. Reactive oxygen species such as H_2_O_2_ can severely damage the photosynthetic apparatus, resulting in lipid peroxidation and protein degradation [67]. Therefore, H_2_O_2_, MDA, and soluble protein concentrations would be interesting indicators of PGPB-induced plant resistance to harsh abiotic environments (e.g., high temperature and drought stress, commons in tropical agriculture), acting on intracellular ROS elimination, decreased membrane peroxidation, stabilization of membrane permeability, and enhancement of plant photosynthesis [20, 50]. In addition, soluble protein plays an essential role in various physiological and metabolic processes within plants, including enzyme catalysis, signal transduction, and stress responses [68]. The higher levels of soluble proteins in co-inoculated treatments suggest improved protein synthesis and potentially enhanced cellular functioning, contributing to the overall growth and performance of corn plants. Moreover, the non-linear response of H_2_O_2_ and MDA to N-application rates without reaching a maximum point (plateau) (Table 2) suggests that the physiological responses were not saturated within the range of N-application rates employed.

### Environment benefits of adding *A. brasilense* and *B. subtilis* to a nitrogen management plan

Based on our results, the potential environmental benefits of partially replacing chemical fertilizers with microbial consortia, such as *A. brasilense* + *B. subtilis*, in corn cultivation can have a significant impact on the entire agroecosystem. Considering the Intergovernmental Panel on Climate Change - IPCC [69] indication that 1 kg of N-fertilizer applied corresponds to approximately 10.5 kg of CO_2_ ^−e^ (carbon dioxide equivalent), our findings suggest that by replacing 240 to 175 kg N ha^−1^ of N fertilizer (-65 kg N ha^−1^) with the microbial consortia, there would be an avoidance of 682.5 kg CO_2_ ^−e^ ha^−1^. In the context of the 4.5 million ha used for corn production in Brazil during the 2022–2023 season (October to March) [70], this approach could potentially lead to an avoidance of 3.07 million Mg (megagrams) of CO_2_ ^−e^ per year. Evidently this is just one projection, and should be better address, however, the reduction in CO_2_ ^−e^ can be even larger if we consider that the total cultivated area with corn in Brazil at 3 crops season^−1^ comprehends 22 million ha. Given that agriculture is the largest source of greenhouse gas emissions (GHGs) in many agricultural countries, including Brazil, where chemical fertilizers account for approximately 30% of emissions in the agricultural sector, adopting microbial inoculation as a partial replacement for chemical fertilizers presents a strategic approach to mitigate GHG emissions [19].

Partial replacing N-fertilizers by microbial consortia not only can enhances crop productivity but also may reduce the overall environmental footprint of agriculture, helping countries effort towards climate and environmental goals. While the results from the study are promising, it is important to recognize that the effectiveness of microbial consortia, including *Azospirillum* sp. and *Bacillus* sp., may vary under different environmental conditions and agricultural management practices. Therefore, further research is needed to investigate the performance of these PGPBs in various climatic regions and production systems. Subtropical and temperate climates, such as those found in North America, Europe, and Asia, may present different challenges and opportunities for microbial inoculation compared to the tropical environments used in this research. Factors such as soil types, temperature fluctuations, and different crop varieties and rotations may influence the interaction between the PGPBs and the plants, potentially impacting the effectiveness of these inoculations [17, 71]. The specific conditions tested in this study, such as no-till practices, the type of N fertilizer used (in our case urea), and the corn hybrid employed are important factors to consider when interpreting the results. These findings may not be directly applicable to all agricultural settings worldwide.

## Conclusions

The microbial consortia with *A. brasilense* and *B. subtilis* can emerge as a promising approach to enhance N use efficiency and N-recovery in corn crops by a myriad of nutritional, physiological, and biochemical aspects related to corn root and shoot growth, N dynamics, photosynthesis related-parameters, and oxidative stress assessment. The inoculation with *A. brasilense* and *B. subtilis*, especially in combination, enhanced the stomatal conductance of corn plants. Also, the microbial consortia positively influenced the ability of corn plants to assimilate CO_2_ and perform photosynthesis, leading to increased C fixation in corn plants, and enhancing root and shoot growth. Our study projects that it would be possible to reduce N application rates from 240 to 175 kg N ha^−1^ while increasing corn yield by 5.2%. In contrast, the benefits of inoculations on corn physiological, biochemical and nutritional parameters are minimal when associated with high N levels above 200 kg N ha^−1^, dramatically affecting N use efficiency and N-recovery.

Finally, the microbial consortia inoculation approach can contribute to more sustainable and productive agricultural practices while minimizing the environmental impact associated with excessive N-fertilizer application. Also, a balanced advance to N application, considering both the beneficial microorganisms and the specific crop nutrient requirements, is decisive to achieve optimal plant growth and yield. Further research should explore the underlying mechanisms and interactions between N rates and microbial inoculations with a focus placed on improving N management strategies in different crop species and environments.

### Supplementary Information

**Additional file 1: Sup. File 1.** Soil pysico-chemical atributes in 0-0.20m depth before field the field trial beginning. Selvíria, state of Mato Grosso do Sul, Brazil. **Sup. File 2.** Daily rainfall (bar), temperature and reference evapotranspiration (lines) in 2019/20 (A) and 2020/21 (B) during corn cropping season. **Sup. File 3.** Summary of statistical analysis (*p*-values) for corn shoot and root biomass, N shoot accumulation, leaf chlorophyll index (LCI), net photosynthetic rate (A), stomatal conductance (Gs), internal CO_2_ concentration in the substomatal chamber (Ci), transpiration (E), H_2_O_2_, malondialdehyde (MDA), leaf soluble proteins, straw production, grain yield, physiological efficiency, N use efficiency (NUE), apparent fertilizer recovery (AFR), nitrate (NO_3_^-^), ammonium (NH_4_^+^), inorganic (NO_3_^-^ + NH_4_^+^) and total N concentrations in straw and soil affected by inoculations, N rates, years of study and their interactions. **Sup. File 4.** Interaction between inoculations and years on corn shoot biomass. **Sup. File 5.** Interaction between N rates and years on corn shoot biomass. **Sup. File 6.** Interaction between N rates and years on corn root biomass. **Sup. File 7.** Interaction between N rates and years on N shoot accumulation. **Sup. File 8.** Interaction between N rates and years on LCI. **Sup. File 9.** Net photosynthetic rate as a function of years of study. **Sup. File 10.** Stomatal conductance as a function of years of study. **Sup. File 11.** Interaction between inoculations and years on leaf transpiration. **Sup. File 12.** Interaction between N rates and years on leaf transpiration. **Sup. File 13.** Interaction between inoculations and years on leaf H_2_O_2_ concentration. **Sup. File 14.** Interaction between N rates and years on leaf H_2_O_2_ concentration. **Sup. File 15.** Interaction between inoculations and years on leaf MDA concentration. **Sup. File 16.** Interaction between N rates and years on leaf MDA concentration. **Sup. File 17.** Interaction between inoculations and years on leaf soluble proteins concentration. **Sup. File 18.** Interaction between N rates and years on leaf soluble proteins concentration. **Sup. File 19.** Interaction between inoculations and years on corn straw production. **Sup. File 20.** Interaction between N rates and years on corn straw production. **Sup. File 21.** Interaction between N rates and years on corn grain yield. **Sup. File 22.** Interaction between inoculations and years on physiological efficiency. **Sup. File 23.** Interaction between N rates and years on physiological efficiency. **Sup. File 24.** Interaction between N rates and years on NUE. **Sup. File 25.** Interaction between inoculations and years on AFR. **Sup. File 26.** Interaction between N rates and years on AFR.

## Data Availability

The data sets generated during this study are available from the corresponding author on reasonable request.
