# Supplementary material for: Impact of nitrogen fertilizer sustainability on corn crop yield: the role of beneficial microbial inoculation interactions
Source: BMC Plant Biol. 2024 Apr 11;24:268. doi: 10.1186/s12870-024-04971-3 (PMC11008049; doi:10.1186/s12870-024-04971-3)
Supplement: Supplementary file 1 — Additional file 1: Sup. File 1. Soil pysico-chemical atributes in 0-0.20m depth before field the field trial beginning. Selvíria, state of Mato Grosso do Sul, Brazil. Sup. File 2. Daily rainfall (bar), temperature and reference evapotranspiration (lines) in 2019/20 (A) and 2020/21 (B) during corn cropping season. Sup. File 3. Summary of statistical analysis (p-values) for corn shoot and root biomass, N shoot accumulation, leaf chlorophyll index (LCI), net photosynthetic rate (A), stomatal conductance (Gs), internal CO2 concentration in the substomatal chamber (Ci), transpiration (E), H2O2, malondialdehyde (MDA), leaf soluble proteins, straw production, grain yield, physiological efficiency, N use efficiency (NUE), apparent fertilizer recovery (AFR), nitrate (NO3-), ammonium (NH4+), inorganic (NO3- + NH4+) and total N concentrations in straw and soil affected by inoculations, N rates, years of study and their interactions. Sup. File 4. Interaction between inoculations and years on corn shoot biomass. Sup. File 5. Interaction between N rates and years on corn shoot biomass. Sup. File 6. Interaction between N rates and years on corn root biomass. Sup. File 7. Interaction between N rates and years on N shoot accumulation. Sup. File 8. Interaction between N rates and years on LCI. Sup. File 9. Net photosynthetic rate as a function of years of study. Sup. File 10. Stomatal conductance as a function of years of study. Sup. File 11. Interaction between inoculations and years on leaf transpiration. Sup. File 12. Interaction between N rates and years on leaf transpiration. Sup. File 13. Interaction between inoculations and years on leaf H2O2 concentration. Sup. File 14. Interaction between N rates and years on leaf H2O2 concentration. Sup. File 15. Interaction between inoculations and years on leaf MDA concentration. Sup. File 16. Interaction between N rates and years on leaf MDA concentration. Sup. File 17. Interaction between inoculations and years on leaf soluble proteins [file 12870_2024_4971_MOESM1_ESM.docx]

Journal: BMC Plant Biology

**Impact of nitrogen fertilizer sustainability on corn crop yield: The role of beneficial microbial inoculation interactions**

Fernando Shintate Galindo^a*^, Paulo Humberto Pagliari^b^, Edson Cabral da Silva^c^, Bruno Horschut de Lima^c^, Guilherme Carlos Fernandes^c^, Cassio Carlette Thiengo^d^, João Victor Silva Bernardes^d^, Arshad Jalal^e^, Carlos Eduardo Silva Oliveira^c^, Lucila de Sousa Vilela^a^, Enes Furlani Junior^c^, Thiago Assis Rodrigues Nogueira^f^, Vagner do Nascimento^a^, Marcelo Carvalho Minhoto Teixeira Filho^c^, José Lavres^d^

^a^ São Paulo State University, College of Agricultural and Technological Sciences, Department of Crop Production, Dracena, 17900-000, Brazil.

^b^ University of Minnesota, Southwest Research and Outreach Center, Department of Soil, Water, and Climate, Lamberton, MN 56152, United States of America.

^c^ São Paulo State University, Department of Plant Health, Rural Engineering, and Soils, Ilha Solteira, 15345-000, Brazil.

^d^ University of São Paulo, Center for Nuclear Energy in Agriculture, Piracicaba, 13416-000, Brazil

^e^ King Abdullah University of Science and Technology, Thuwal, 23955-6900, Kingdom of Saudi Arabia.

^f^ São Paulo State University, Department of Agricultural Sciences, Jaboticabal, 14884-900, Brazil.

*** Corresponding author:** fernando.galindo@unesp.br (FS Galindo)

Mailing address: São Paulo State University, College of Agricultural and Technological Sciences, Rod. Cmte João Ribeiro de Barros, km 651 - Bairro das Antas, ZIP Code: 17900-000, Dracena, Brazil.

Phone number: +55 18 98120 8054

**Sup. File 1.** Soil pysico-chemical atributes in 0-0.20m depth before field the field trial beginning. Selvíria, state of Mato Grosso do Sul, Brazil.

| **Soil chemical attributes** | **Unity** | **0-0.20m** |
| --- | --- | --- |
| Total N | g kg^-1^ | 0.62 ± 0.007 |
| N-NO_3_^-^ | mg kg^-1^ | 76.4 ± 8.8 |
| N-NH_4_^+^ | mg kg^-1^ | 59.9 ± 4.8 |
| P (resin) | mg dm^-3^ | 20 ± 2.7 |
| S (SO_4_) | mg dm^-3^ | 4 ± 1.4 |
| Organic matter | g dm^-3^ | 23 ± 2.8 |
| pH (CaCl_2_) |  | 5.4 ± 0.14 |
| K (resin) | mmol_c_ dm^-3^ | 2.1 ± 0.07 |
| Ca (resin) | mmol_c_ dm^-3^ | 20 ± 0.71 |
| Mg (resin) | mmol_c_ dm^-3^ | 17 ± 0.71 |
| H+Al | mmol_c_ dm^-3^ | 23 ± 3.5 |
| Al | mmol_c_ dm^-3^ | 0 ± 0.001 |
| B (hot water) | mg dm^-3^ | 0.03 ± 0.01 |
| Cu (DTPA) | mg dm^-3^ | 2.9 ± 0.21 |
| Fe (DTPA) | mg dm^-3^ | 18 ± 0.71 |
| Mn (DTPA) | mg dm^-3^ | 19 ± 1.5 |
| Zn (DTPA) | mg dm^-3^ | 0.8 ± 0.07 |
| Cation exchange capacity (pH 7.0) | mmol_c_ dm^-3^ | 61 ± 3.5 |
| Base saturation | % | 63 ± 4.2 |
| **Granulometry** | **Unity** | **0-0.20m** |
| Clay | g kg^-1^ | 425 ± 4.2 |
| Sand | g kg^-1^ | 534 ± 2.8 |
| Silt | g kg^-1^ | 41 ± 1.4 |

*n* = 20; ± refers to standard deviation of the mean;

Total N: Semi-micro Kjeldahl method. pH: active acidity in CaCl_2_ (0.01 mol L^-1^). O.M.: organic matter by the dichromate/colorimetric method. P, K, Ca e Mg: extracted by ion exchange resin. Al: exchangeable aluminum by titration. H+Al: Potential acidity by pH SMP. S: (S-SO_4_)^-2^ extracted by Ca(H_2_PO_4_)_2_ 0.01 mol L^-1^ determined by turbidimetry. Cu, Fe, Mn and Zn: DTPA = diethylenetriaminepentaacetic acid.

**Sup. File 2.** Daily rainfall (bar), temperature and reference evapotranspiration (lines) in 2019/20 (A) and 2020/21 (B) during corn cropping season.

| **A.** |
| --- |
|  |

T max, T min and ETo refers to maximum and minimum temperatures, and ETo to reference evapotranspiration, respectively.

**Sup. File 3.** Summary of statistical analysis (*p*-values) for corn shoot and root biomass, N shoot accumulation, leaf chlorophyll index (LCI), net photosynthetic rate (A), stomatal conductance (Gs), internal CO_2_ concentration in the substomatal chamber (Ci), transpiration (E), H_2_O_2_, malondialdehyde (MDA), leaf soluble proteins, straw production, grain yield, physiological efficiency, N use efficiency (NUE), apparent fertilizer recovery (AFR), nitrate (NO_3_^-^), ammonium (NH_4_^+^), inorganic (NO_3_^-^ + NH_4_^+^) and total N concentrations in straw and soil affected by inoculations, N rates, years of study and their interactions.

| ***p*-value** | **Shoot biomass** | **Root biomass** | **N shoot accumulation** | **LCI** | **A** | **Gs** | **Ci** | **E** |
| --- | --- | --- | --- | --- | --- | --- | --- | --- |
| Ino (I) | 0.001 | 0.001 | 0.001 | 0.001 | 0.001 | 0.001 | 0.108 | 0.001 |
| Rates (R) | 0.001 | 0.001 | 0.001 | 0.001 | 0.001 | 0.001 | 0.320 | 0.030 |
| Year (Y) | 0.001 | 0.001 | 0.008 | 0.001 | **0.001** | **0.003** | 0.969 | 0.001 |
| I × R | **0.003** | **0.002** | **0.003** | **0.004** | **0.001** | **0.013** | **0.001** | **0.001** |
| I × Y | **0.002** | 0.154 | 0.109 | 0.852 | 0.689 | 0.846 | 0.657 | **0.001** |
| R × Y | **0.009** | **0.007** | **0.001** | **0.001** | 0.190 | 0.055 | 0.183 | **0.044** |
| I × R × Y | 0.057 | 0.327 | 0.126 | 0.809 | 0.452 | 0.109 | 0.313 | 0.609 |
| ***p*-value** | **H_2_O_2_** | **MDA** | **Soluble proteins** | **Straw** | **Grain yield** | **Physiological efficiency** | **NUE** | **AFR** |
| Ino (I) | 0.001 | 0.001 | 0.001 | 0.001 | 0.001 | 0.001 | 0.001 | 0.001 |
| Rates (R) | 0.001 | 0.001 | 0.001 | 0.001 | 0.001 | 0.001 | 0.001 | 0.001 |
| Year (Y) | 0.001 | 0.001 | 0.001 | 0.001 | 0.001 | 0.001 | 0.001 | 0.001 |
| I × R | **0.016** | **0.008** | **0.001** | **0.018** | **0.004** | **0.001** | **0.001** | **0.001** |
| I × Y | **0.002** | **0.002** | **0.007** | **0.016** | 0.107 | **0.002** | 0.114 | **0.007** |
| R × Y | **0.002** | **0.002** | **0.001** | **0.002** | **0.001** | **0.002** | **0.001** | **0.002** |
| I × R × Y | 0.725 | 0.127 | 0.417 | 0.104 | 0.066 | 0.088 | 0.125 | 0.236 |
| ***p*-value** | **N-NO_3_^-^ straw** | **N-NH_4_^+^ straw** | **N-NO_3_^-^ + N-NH_4_^+^ straw** | **Total N straw** | **N-NO_3_^-^ soil** | **N-NH_4_^+^ soil** | **N-NO_3_^-^ + N-NH_4_^+^ soil** | **Total N soil** |
| Ino (I) | **0.006** | **0.001** | **0.001** | **0.001** | **0.001** | **0.032** | 0.095 | 0.065 |
| Rates (R) | - | - | - | - | - | - | - | - |
| Year (Y) | - | - | - | - | - | - | - | - |
| I × R | - | - | - | - | - | - | - | - |
| I × Y | - | - | - | - | - | - | - | - |
| R × Y | - | - | - | - | - | - | - | - |
| I × R × Y | - | - | - | - | - | - | - | - |

**Sup. File 4.** Interaction between inoculations and years on corn shoot biomass.

| **Shoot biomass (kg ha^-1^)** | | |
| --- | --- | --- |
| I × Y | 1 | 2 |
| Control | 9382 ± 630 cA | 9116 ± 842 cB |
| Azo | 10077 ± 1068 bA | 9564 ± 432 bB |
| Bac | 10549 ± 760 aA | 9600 ± 788 bB |
| Azo+Bac | 10291 ± 989 bA | 10059 ± 609 aB |
| L.S.D. (5%) I | 227 |  |
| L.S.D. (5%) Y | 172 |  |

Lowercase letters represent the difference between inoculations at each year of study. Uppercase letters represent the difference between years at each inoculation tested; L.S.D. = Least significant difference; Ctl = control, Azo = single inoculation with *A. brasilense*, Bac = single inoculation with *B. subtilis* and Azo+Bac = co-inoculation with *A. brasilense* and *B. subtilis*; ± refers to standard deviation of the mean

**Sup. File 5.** Interaction between N rates and years on corn shoot biomass.

| **Shoot biomass (kg ha^-1^)** | | | | | |
| --- | --- | --- | --- | --- | --- |
| R × Y | 0 | 60 | 120 | 180 | 240 |
| 1 | 8847 ± 898 a | 9903 ± 768 a | 10337 ± 942 a | 10950 ± 1043 a | 10337 ± 536 a |
| 2 | 8836 ± 958 a | 9438 ± 534 b | 10094 ± 542 b | 9968 ± 761 b | 9588 ± 509 b |
| L.S.D. (5%) | 193 |  |  |  |  |

1 Ŷ = 8818.938 + 21.747x - 0.062x^2^ (R^2^ = 0.96^**^)

2 Ŷ = 8786.610 + 16.463x - 0.054x^2^ (R^2^ = 0.96^**^)

Different letters indicate a difference between means, according to the Tukey test (*p* < 0.05); L.S.D. = Least significant difference. ± refers to standard deviation of the mean.

**Sup. File 6.** Interaction between N rates and years on corn root biomass.

| **Root biomass (kg ha^-1^)** | | | | | |
| --- | --- | --- | --- | --- | --- |
| R × Y | 0 | 60 | 120 | 180 | 240 |
| 1 | 636 ± 137 b | 858 ± 120 a | 900 ± 123 b | 1044 ± 133 a | 896 ± 118 b |
| 2 | 759 ± 143 a | 882 ± 133 a | 1113 ± 117 a | 1024 ± 106 a | 962 ± 136 a |
| L.S.D. (5%) | 50.41 |  |  |  |  |

1 Ŷ = 634.785 + 4.218x - 0.012x^2^ (R^2^ = 0.91^**^)

2 Ŷ = 739.985 + 4.197x - 0.013x^2^ (R^2^ = 0.88^**^)

Different letters indicate a difference between means, according to the Tukey test (*p* < 0.05); L.S.D. = Least significant difference. ± refers to standard deviation of the mean.

**Sup. File 7.** Interaction between N rates and years on N shoot accumulation.

| **N shoot accumulation (kg ha^-1^)** | | | | | |
| --- | --- | --- | --- | --- | --- |
| R × Y | 0 | 60 | 120 | 180 | 240 |
| 1 | 117.2 ± 11.2 a | 177.5 ± 16.4 a | 184.3 ± 14.2 a | 204.5 ± 16.5 a | 184.5 ± 17.3 a |
| 2 | 92.7 ± 9.1 b | 106.3 ± 9.2 b | 120.4 ± 12.1 b | 121.0 ± 13.5 b | 108.0 ± 10.3 b |
| L.S.D. (5%) | 11.70 |  |  |  |  |

1 Ŷ = 120.286 + 0.970x - 0.002x^2^ (R^2^ = 0.95^**^)

2 Ŷ = 91.117 + 0.393x - 0.001x^2^ (R^2^ = 0.96^**^)

Different letters indicate a difference between means, according to the Tukey test (*p* < 0.05); L.S.D. = Least significant difference. ± refers to standard deviation of the mean.

**Sup. File 8.** Interaction between N rates and years on LCI.

| **LCI** | | | | | |
| --- | --- | --- | --- | --- | --- |
| R × Y | 0 | 60 | 120 | 180 | 240 |
| 1 | 67.93 ± 2.4 a | 69.06 ± 3.7 a | 70.27 ± 4.0 b | 69.11 ± 3.2 b | 68.77 ± 2.8 b |
| 2 | 64.52 ± 5.2 b | 69.84 ± 4.3 a | 73.59 ± 4.6 a | 73.95 ± 4.3 a | 72.96 ± 3.9 a |
| L.S.D. (5%) | 2.40 |  |  |  |  |

1 Ŷ = 67.929 + 0.028x - 0.0001x^2^ (R^2^ = 0.82^*^)

2 Ŷ = 64.490 + 0.111x - 0.0003x^2^ (R^2^ = 0.99^**^)

Different letters indicate a difference between means, according to the Tukey test (*p* < 0.05); L.S.D. = Least significant difference. ± refers to standard deviation of the mean.

**Sup. File 9.** Net photosynthetic rate as a function of years of study.

| **A (µmol CO_2_ m^-2^ s^-1^)** | |
| --- | --- |
| Year | |
| 1 | 28.10 ± 2.9 a |
| 2 | 26.56 ± 2.7 b |
| L.S.D. (5%) | 0.80 |

Different letters indicate a difference between means, according to the Tukey test (*p* < 0.05); L.S.D. = Least significant difference. ± refers to standard deviation of the mean.

**Sup. File 10.** Stomatal conductance as a function of years of study.

| **Gs (mol H_2_O m^-2^ s^-1^)** | |
| --- | --- |
| Year | |
| 1 | 0.55 ± 0.05 a |
| 2 | 0.49 ± 0.03 b |
| L.S.D. (5%) | 0.03 |

Different letters indicate a difference between means, according to the Tukey test (*p* < 0.05); L.S.D. = Least significant difference. ± refers to standard deviation of the mean.

**Sup. File 11.** Interaction between inoculations and years on leaf transpiration.

| **E (mmol H_2_O m^-2^ s^-1^)** | | |
| --- | --- | --- |
| I × Y | 1 | 2 |
| Control | 2.40 ± 0.2 bA | 2.05 ± 0.2 bB |
| Azo | 2.83 ± 0.3 aA | 2.13 ± 0.3 bB |
| Bac | 2.39 ± 0.2 bA | 2.47 ± 0.3 aA |
| Azo+Bac | 2.30 ± 0.4 bA | 2.41 ± 0.2 aA |
| L.S.D. (5%) I | 0.18 |  |
| L.S.D. (5%) Y | 0.14 |  |

Lowercase letters represent the difference between inoculations at each year of study. Uppercase letters represent the difference between years at each inoculation tested; L.S.D. = Least significant difference; Ctl = control, Azo = single inoculation with *A. brasilense*, Bac = single inoculation with *B. subtilis* and Azo+Bac = co-inoculation with *A. brasilense* and *B. subtilis*. ± refers to standard deviation of the mean.

**Sup. File 12.** Interaction between N rates and years on leaf transpiration.

| **E (mmol H_2_O m^-2^ s^-1^)** | | | | | |
| --- | --- | --- | --- | --- | --- |
| R × Y | 0 | 60 | 120 | 180 | 240 |
| 1 | 2.47 ± 0.2 a | 2.37 ± 0.3 a | 2.51 ± 0.3 a | 2.56 ± 0.2 a | 2.48 ± 0.3 a |
| 2 | 2.24 ± 0.2 b | 2.21 ± 0.2 b | 2.31 ± 0.2 b | 2.14 ± 0.3 b | 2.42 ± 0.2 a |
| L.S.D. (5%) | 0.16 |  |  |  |  |

1 ns

2 ns

Different letters indicate a difference between means, according to the Tukey test (*p* < 0.05); L.S.D. = Least significant difference; ns = not significant. ± refers to standard deviation of the mean.

**Sup. File 13.** Interaction between inoculations and years on leaf H_2_O_2_ concentration.

| **H_2_O_2_ (µmol g^-1^ F.M.)** | | |
| --- | --- | --- |
| I × Y | 1 | 2 |
| Control | 4607 ± 124 aB | 4884 ± 151 aA |
| Azo | 4336 ± 366 bB | 4709 ± 302 bA |
| Bac | 4211 ± 190 bcB | 4615 ± 155 abA |
| Azo+Bac | 4165 ± 343 cB | 4559 ± 311 bA |
| L.S.D. (5%) I | 134.9 |  |
| L.S.D. (5%) Y | 102.6 |  |

Lowercase letters represent the difference between inoculations at each year of study. Uppercase letters represent the difference between years at each inoculation tested; L.S.D. = Least significant difference; Ctl = control, Azo = single inoculation with *A. brasilense*, Bac = single inoculation with *B. subtilis* and Azo+Bac = co-inoculation with *A. brasilense* and *B. subtilis*. ± refers to standard deviation of the mean.

**Sup. File 14.** Interaction between N rates and years on leaf H_2_O_2_ concentration.

| **H_2_O_2_ (µmol g^-1^ F.M.)** | | | | | |
| --- | --- | --- | --- | --- | --- |
| R × Y | 0 | 60 | 120 | 180 | 240 |
| 1 | 4536 ± 259 b | 4371 ± 288 b | 4151 ± 168 b | 4093 ± 247 b | 4500 ± 158 b |
| 2 | 4899 ± 335 a | 4793 ± 177 a | 4523 ± 180 a | 4478 ± 133 a | 4765 ± 186 a |
| L.S.D. (5%) | 114.8 |  |  |  |  |

1 Ŷ = 4586.911 - 6.796x + 0.025x^2^ (R^2^ = 0.83^**^)

2 Ŷ = 4952.902 - 5.781x + 0.020x^2^ (R^2^ = 0.80^**^)

Different letters indicate a difference between means, according to the Tukey test (*p* < 0.05); L.S.D. = Least significant difference. ± refers to standard deviation of the mean.

**Sup. File 15.** Interaction between inoculations and years on leaf MDA concentration.

| **MDA (nmol g^-1^ F.M.)** | | |
| --- | --- | --- |
| I × Y | 1 | 2 |
| Control | 16.72 ± 1.3 aB | 31.26 ± 2.6 aA |
| Azo | 16.64 ± 1.4 aB | 30.33 ± 2.7 aA |
| Bac | 16.31 ± 1.6 aB | 29.01 ± 2.6 bA |
| Azo+Bac | 16.24 ± 1.4 aB | 28.36 ± 2.3 bA |
| L.S.D. (5%) I | 1.07 |  |
| L.S.D. (5%) Y | 0.81 |  |

Lowercase letters represent the difference between inoculations at each year of study. Uppercase letters represent the difference between years at each inoculation tested; L.S.D. = Least significant difference; Ctl = control, Azo = single inoculation with *A. brasilense*, Bac = single inoculation with *B. subtilis* and Azo+Bac = co-inoculation with *A. brasilense* and *B. subtilis*. ± refers to standard deviation of the mean.

**Sup. File 16.** Interaction between N rates and years on leaf MDA concentration.

| **MDA (nmol g^-1^ F.M.)** | | | | | |
| --- | --- | --- | --- | --- | --- |
| R × Y | 0 | 60 | 120 | 180 | 240 |
| 1 | 17.16 ± 1.6 b | 16.77 ± 1.5 b | 14.14 ± 0.6 b | 17.07 ± 1.2 b | 17.24 ± 1.3 b |
| 2 | 42.57 ± 3.1 a | 31.46 ± 2.8 a | 23.20 ± 2.3 a | 23.44 ± 2.4 a | 28.03 ± 2.8 a |
| L.S.D. (5%) | 0.91 |  |  |  |  |

1 Ŷ = 17.340 - 0.031x + 0.0001x^2^ (R^2^ = 0.46^**^)

2 Ŷ = 42.860 - 0.251x + 0.0007x^2^ (R^2^ = 0.99^**^)

Different letters indicate a difference between means, according to the Tukey test (*p* < 0.05); L.S.D. = Least significant difference. ± refers to standard deviation of the mean.

**Sup. File 17.** Interaction between inoculations and years on leaf soluble proteins concentration.

| **Soluble proteins (mg mL^-1^)** | | |
| --- | --- | --- |
| I × Y | 1 | 2 |
| Control | 1.26 ± 0.1 cB | 1.48 ± 0.2 cA |
| Azo | 1.32 ± 0.2 bcB | 1.54 ± 0.2 bA |
| Bac | 1.32 ± 0.2 bB | 1.56 ± 0.2 abA |
| Azo+Bac | 1.42 ± 0.2 aB | 1.61 ± 0.1 aA |
| L.S.D. (5%) I | 0.06 |  |
| L.S.D. (5%) Y | 0.04 |  |

Lowercase letters represent the difference between inoculations at each year of study. Uppercase letters represent the difference between years at each inoculation tested; L.S.D. = Least significant difference; Ctl = control, Azo = single inoculation with *A. brasilense*, Bac = single inoculation with *B. subtilis* and Azo+Bac = co-inoculation with *A. brasilense* and *B. subtilis*. ± refers to standard deviation of the mean.

**Sup. File 18.** Interaction between N rates and years on leaf soluble proteins concentration.

| **Soluble proteins (mg mL^-1^)** | | | | | |
| --- | --- | --- | --- | --- | --- |
| R × Y | 0 | 60 | 120 | 180 | 240 |
| 1 | 0.96 ± 0.1 b | 1.40 ± 0.1 a | 1.63 ± 0.2 b | 1.42 ± 0.2 b | 1.24 ± 0.1 b |
| 2 | 1.12 ± 0.2 a | 1.36 ± 0.2 a | 1.75 ± 0.2 a | 1.75 ± 0.1 a | 1.74 ± 0.1 a |
| L.S.D. (5%) | 0.05 |  |  |  |  |

1 Ŷ = 0.983 + 0.008x - 0.00003x^2^ (R^2^ = 0.95^**^)

2 Ŷ = 1.097 + 0.006x - 0.00001x^2^ (R^2^ = 0.96^**^)

Different letters indicate a difference between means, according to the Tukey test (*p* < 0.05); L.S.D. = Least significant difference. ± refers to standard deviation of the mean.

**Sup. File 19.** Interaction between inoculations and years on corn straw production.

| **Straw (kg ha^-1^)** | | |
| --- | --- | --- |
| I × Y | 1 | 2 |
| Control | 9210 ± 551 cA | 7654 ± 764 bB |
| Azo | 9891 ± 641 bA | 8497 ± 731 aB |
| Bac | 10355 ± 933 aA | 8395 ± 838 aB |
| Azo+Bac | 10102 ± 730 abA | 8712 ± 827 aB |
| L.S.D. (5%) I | 370.8 |  |
| L.S.D. (5%) Y | 281.7 |  |

Lowercase letters represent the difference between inoculations at each year of study. Uppercase letters represent the difference between years at each inoculation tested; L.S.D. = Least significant difference; Ctl = control, Azo = single inoculation with *A. brasilense*, Bac = single inoculation with *B. subtilis* and Azo+Bac = co-inoculation with *A. brasilense* and *B. subtilis*. ± refers to standard deviation of the mean.

**Sup. File 20.** Interaction between N rates and years on corn straw production.

| **Straw (kg ha^-1^)** | | | | | |
| --- | --- | --- | --- | --- | --- |
| R × Y | 0 | 60 | 120 | 180 | 240 |
| 1 | 8685 ± 270 a | 9721 ± 573 a | 10147 ± 625 a | 10749 ± 836 a | 10147 ± 476 a |
| 2 | 7270 ± 407 b | 7875 ± 460 b | 9048 ± 865 b | 8851 ± 440 b | 8529 ± 774 b |
| L.S.D. (5%) | 314.9 |  |  |  |  |

1 Ŷ = 8656.915 + 21.348x - 0.061x^2^ (R^2^ = 0.96^**^)

2 Ŷ = 7155.942 + 21.167x - 0.063x^2^ (R^2^ = 0.91^**^)

Different letters indicate a difference between means, according to the Tukey test (*p* < 0.05); L.S.D. = Least significant difference. ± refers to standard deviation of the mean.

**Sup. File 21.** Interaction between N rates and years on corn grain yield.

| **Grain yield (kg ha^-1^)** | | | | | |
| --- | --- | --- | --- | --- | --- |
| R × Y | 0 | 60 | 120 | 180 | 240 |
| 1 | 8163 ± 669 a | 9435 ± 777 a | 10028 ± 583 a | 10386 ± 892 a | 10181 ± 585 a |
| 2 | 6915 ± 701 b | 7699 ± 707 b | 7828 ± 774 b | 8166 ± 685 b | 7885 ± 700 b |
| L.S.D. (5%) | 401.7 |  |  |  |  |

1 Ŷ = 8186.368 + 23.490x - 0.063x^2^ (R^2^ = 0.99^**^)

2 Ŷ = 6943.139 + 13.153x - 0.038x^2^ (R^2^ = 0.95^**^)

Different letters indicate a difference between means, according to the Tukey test (*p* < 0.05); L.S.D. = Least significant difference. ± refers to standard deviation of the mean.

**Sup. File 22.** Interaction between inoculations and years on physiological efficiency.

| **Physiological efficiency (kg grain kg N accumulated^-1^)** | | |
| --- | --- | --- |
| I × Y | 1 | 2 |
| Control | 56.39 ± 5.2 aB | 70.03 ± 6.1 aA |
| Azo | 58.29 ± 5.4 aA | 58.04 ± 5.8 bA |
| Bac | 54.69 ± 5.1 aB | 75.20 ± 6.3 aA |
| Azo+Bac | 45.66 ± 4.3 bB | 57.92 ± 5.6 bA |
| L.S.D. (5%) I | 7.59 |  |
| L.S.D. (5%) Y | 5.76 |  |

Lowercase letters represent the difference between inoculations at each year of study. Uppercase letters represent the difference between years at each inoculation tested; L.S.D. = Least significant difference; Ctl = control, Azo = single inoculation with *A. brasilense*, Bac = single inoculation with *B. subtilis* and Azo+Bac = co-inoculation with *A. brasilense* and *B. subtilis*. ± refers to standard deviation of the mean.

**Sup. File 23.** Interaction between N rates and years on physiological efficiency.

| **Physiological efficiency (kg grain kg N accumulated^-1^)** | | | | |
| --- | --- | --- | --- | --- |
| R × Y | 60 | 120 | 180 | 240 |
| 1 | 76.49 ± 7.2 b | 53.00 ± 6.2 a | 44.78 ±4.6 b | 40.76 ± 4.1 b |
| 2 | 98.87 ± 8.4 a | 58.00 ± 6.1 a | 55.31 ± 5.5 a | 49.02 ± 5.3 a |
| L.S.D. (5%) | 5.76 |  |  |  |

1 Ŷ = 106.960 - 0.598x + 0.001x^2^ (R^2^ = 0.99^**^)

2 Ŷ = 146.588 - 0.974x + 0.002x^2^ (R^2^ = 0.94^**^)

Different letters indicate a difference between means, according to the Tukey test (*p* < 0.05); L.S.D. = Least significant difference. ± refers to standard deviation of the mean.

**Sup. File 24.** Interaction between N rates and years on NUE.

| **NUE (kg grain kg N applied^-1^)** | | | | |
| --- | --- | --- | --- | --- |
| R × Y | 60 | 120 | 180 | 240 |
| 1 | 40.58 ± 4.0 a | 23.78 ± 3.4 a | 17.84 ± 3.2 a | 12.52 ± 2.0 a |
| 2 | 31.62 ± 3.2 b | 16.67 ± 1.8 b | 12.99 ± 2.0 b | 8.57 ± 1.5 b |
| L.S.D. (5%) | 3.27 |  |  |  |

1 Ŷ = 46.213 - 0.150x (R^2^ = 0.91^**^)

2 Ŷ = 35.671 - 0.121x (R^2^ = 0.88^**^)

Different letters indicate a difference between means, according to the Tukey test (*p* < 0.05); L.S.D. = Least significant difference. ± refers to standard deviation of the mean.

**Sup. File 25.** Interaction between inoculations and years on AFR.

| **AFR (%)** | | |
| --- | --- | --- |
| I × Y | 1 | 2 |
| Control | 33.36 ± 3.2 cA | 17.03 ± 1.7 bB |
| Azo | 44.81 ± 3.8 bA | 30.56 ± 3.2 aB |
| Bac | 47.36 ± 4.1 abA | 20.74 ± 1.8 bB |
| Azo+Bac | 53.72 ± 4.8 aA | 34.26 ± 3.2 aB |
| L.S.D. (5%) I | 6.88 |  |
| L.S.D. (5%) Y | 5.22 |  |

Lowercase letters represent the difference between inoculations at each year of study. Uppercase letters represent the difference between years at each inoculation tested; L.S.D. = Least significant difference; Ctl = control, Azo = single inoculation with *A. brasilense*, Bac = single inoculation with *B. subtilis* and Azo+Bac = co-inoculation with *A. brasilense* and *B. subtilis*. ± refers to standard deviation of the mean.

**Sup. File 26.** Interaction between N rates and years on AFR.

| **AFR (%)** | | | | |
| --- | --- | --- | --- | --- |
| R × Y | 60 | 120 | 180 | 240 |
| 1 | 58.69 ± 5.7 a | 50.64 ± 5.1 a | 38.96 ± 4.0 a | 30.97 ± 3.2 a |
| 2 | 26.78 ± 3.1 b | 32.21 ± 3.0 b | 23.77 ± 2.5 b | 19.84 ± 1.6 b |
| L.S.D. (5%) | 5.22 |  |  |  |

1 Ŷ = 68.530 - 0.158x (R^2^ = 0.99^**^)

2 Ŷ = 21.267 + 0.146x - 0.0006x^2^ (R^2^ = 0.79^**^)

Different letters indicate a difference between means, according to the Tukey test (*p* < 0.05); L.S.D. = Least significant difference. ± refers to standard deviation of the mean.
